# Resistance of mitochondrial DNA to cadmium and Aflatoxin B_1_ damage-induced germline mutation accumulation in *C. elegans*

**DOI:** 10.1093/nar/gkac666

**Published:** 2022-08-10

**Authors:** Tess C Leuthner, Laura Benzing, Brendan F Kohrn, Christina M Bergemann, Michael J Hipp, Kathleen A Hershberger, Danielle F Mello, Tymofii Sokolskyi, Kevin Stevenson, Ilaria R Merutka, Sarah A Seay, Simon G Gregory, Scott R Kennedy, Joel N Meyer

**Affiliations:** Nicholas School of the Environment, Duke University, Durham, NC 27708, USA; Nicholas School of the Environment, Duke University, Durham, NC 27708, USA; Department of Laboratory Medicine and Pathology, University of Washington, Seattle, WA 98195, USA; Nicholas School of the Environment, Duke University, Durham, NC 27708, USA; Department of Laboratory Medicine and Pathology, University of Washington, Seattle, WA 98195, USA; Nicholas School of the Environment, Duke University, Durham, NC 27708, USA; Nicholas School of the Environment, Duke University, Durham, NC 27708, USA; Nicholas School of the Environment, Duke University, Durham, NC 27708, USA; Duke Molecular Physiology Institute, Duke University, Durham, NC 27701, USA; Nicholas School of the Environment, Duke University, Durham, NC 27708, USA; Nicholas School of the Environment, Duke University, Durham, NC 27708, USA; Duke Molecular Physiology Institute, Duke University, Durham, NC 27701, USA; Department of Neurology, Duke University, Durham, NC 27708, USA; Department of Laboratory Medicine and Pathology, University of Washington, Seattle, WA 98195, USA; Nicholas School of the Environment, Duke University, Durham, NC 27708, USA

## Abstract

Mitochondrial DNA (mtDNA) is prone to mutation in aging and over evolutionary time, yet the processes that regulate the accumulation of *de novo* mtDNA mutations and modulate mtDNA heteroplasmy are not fully elucidated. Mitochondria lack certain DNA repair processes, which could contribute to polymerase error-induced mutations and increase susceptibility to chemical-induced mtDNA mutagenesis. We conducted error-corrected, ultra-sensitive Duplex Sequencing to investigate the effects of two known nuclear genome mutagens, cadmium and Aflatoxin B_1_, on germline mtDNA mutagenesis in *Caenorhabditis elegans*. Detection of thousands of mtDNA mutations revealed pervasive heteroplasmy in *C. elegans* and that mtDNA mutagenesis is dominated by C:G → A:T mutations generally attributed to oxidative damage. However, there was no effect of either exposure on mtDNA mutation frequency, spectrum, or trinucleotide context signature despite a significant increase in nuclear mutation rate after aflatoxin B_1_ exposure. Mitophagy-deficient mutants *pink-1* and *dct-1* accumulated significantly higher levels of mtDNA damage compared to wild-type *C. elegans* after exposures. However, there were only small differences in mtDNA mutation frequency, spectrum, or trinucleotide context signature compared to wild-type after 3050 generations, across all treatments. These findings suggest mitochondria harbor additional previously uncharacterized mechanisms that regulate mtDNA mutational processes across generations.

## INTRODUCTION

Mitochondria are vital organelles that provide energy and important signaling molecules for almost every form of eukaryotic life on earth. Phylogenetic evidence strongly suggests that mitochondria share ancestry with alpha-proteobacteria and arose by endosymbiosis. Over the course of 1.45 billion years of coevolution, most genes encoding mitochondrial proteins have translocated into the nuclear genome. Nevertheless, mitochondria still maintain a unique genome that encodes various electron transport chain subunit proteins that are essential for energy production (1).

mtDNA mutations are implicated in diseases affecting at least one person in 5000 (2), in addition to potential roles in many neurological and metabolic disorders, various cancers, and aging (3–5). MtDNA mutation rates are often 100 times higher than nuclear DNA mutation rates in aging and over evolutionary time (6,7,8). Currently, replication errors by the sole mitochondrial DNA polymerase, Pol γ, rather than oxidative stress, are thought to be the main source of mtDNA mutations (9–11). Pol γ contains 3’-5’-exonuclease and 5’-deoxyribose lyase activities which allow for proofreading during mtDNA replication and base excision repair (12). Pol γ has limited translesion synthesis capabilities, potentially rendering mtDNA sensitive to damage-induced mutations or copy number changes due to polymerase stalling at bulky adducts (13). Mitochondria have efficient base excision repair mechanisms, but lack nucleotide excision repair and mismatch repair machinery, which may also contribute to higher mutation rates (14).

Chemical exposure can contribute to nuclear DNA mutagenesis, but few studies have investigated the role of genotoxicants in mtDNA mutagenesis. Evidence is limited regarding the effects of exogenous stress on mtDNA damage and somatic mutagenesis (15), and to our knowledge, the effects of chemical exposure on germline mtDNA mutagenesis have yet to be investigated. This is critical to understand because the basic biology of mitochondria renders mtDNA particularly susceptible to the harmful effects of environmental toxicants and stress. The negative charge of the mitochondrial matrix and Ca^2+^ transporters results in accumulation of cationic heavy metal pollutants, and the phospholipid bilayer attracts lipophilic organic pollutants which can be activated to toxic (particularly genotoxic) metabolites by mitochondrial cytochrome P450s (16–18).

Though mitochondria are limited in DNA repair capacity, organelle dynamics play a significant role in eliminating irreparable damage and maintaining mitochondrial homeostasis. The importance of mitochondrial fission, fusion, and mitophagy in regulating mitochondrial quality are highlighted through manifestation of disease phenotypes from mutations in nuclear encoded genes (19). For example, mutations in nuclear mitophagy genes PARK2 and PINK1 are associated with Parkinson's disease. Mitochondrial dynamics are also important for mediating response to mitochondrial stress, whether metabolic or chemical, as we have reviewed in the context of pollutant stress (20). A damaged organelle can fuse with a healthy organelle, and subsequently undergo fission to create healthy daughter mitochondria (‘complementation’). Alternatively, damaged mitochondria may be selectively targeted for degradation via mitophagy. We have previously demonstrated that genetic disruption of these pathways in *Caenorhabditis elegans* results in increased sensitivity to various stressors and inability to remove ultraviolet C radiation-induced mtDNA damage (21–24). Until this study, variation in accumulation of mtDNA damage associated with mitophagy function after chemical exposure had not been investigated.

Mitophagy is also thought to regulate the selective inheritance of mitochondrial genomes in the germline, where mtDNA is, in most metazoans, uniparentally inherited. Despite the high mtDNA copy number in oocytes, only a few mtDNA genome copies are distributed into primordial germ cells, an event that is referred to as the germline mitochondrial ‘bottleneck’ (25). Evolutionary models predict that the bottleneck increases the probability of removal of deleterious mtDNA variants (26). Whether transmission of mutant mitochondrial genomes is stochastic, permitting genetic drift, or targeted via purifying selection, is controversial (27–30).

Previous studies in *C. elegans* have demonstrated that mitophagy can play an important role in regulating the germline transmission of mtDNA variants. For example, progeny of *C. elegans* that lack functional Parkin (*pdr-1*) accumulate higher frequencies of the 3.1 kb *uaDf5* mtDNA deletion compared to wild-type (31). However, the role of mitophagy in regulating the transmission of *de novo* single nucleotide mutations through the germline remains an exciting area of investigation, particularly in the context of exposure to chemicals that cause mtDNA damage. A recent study by Haroon *et al.* suggests that mitophagy may play a role in mtDNA single nucleotide mutagenesis, as *C. elegans* deficient in *pdr-1* accumulate higher frequencies of mtDNA point mutations than wild-type, though only in a Pol γ exonuclease mutant background (32). Therefore, we hypothesized that exposure to mtDNA-damaging chemicals would result in higher rates of mtDNA mutation accumulation, and that this would be exacerbated in mitophagy-deficient *C. elegans*.

*C. elegans* is a well-established organism in which fundamental knowledge of spontaneous mtDNA mutational processes and other evolutionary insights (including genotoxin-induced nuclear mutation studies) has been achieved through classical mutation accumulation line experiments (33). Two independent mutation accumulation line (MA) experiments have been conducted in wild-type *C. elegans*, though only Konrad *et al.* conducted next-generation Illumina sequencing (8,34). A third MA experiment was conducted in the *C. elegans* mutant *gas-1*, which renders complex I of the ETC dysfunctional, thereby resulting in increased reactive oxygen species production and reduced ATP production (35). However, no other MA approach has investigated the effect of either chemical stressors or mitophagy on mtDNA mutagenesis in *C. elegans*.

A limitation of previous MA studies in *C. elegans* is that only a few mtDNA variants that arose to high frequencies or fixation were detected, with one very recent exception (36). However, it is now known that most mtDNA variants are not only heteroplasmic, but exist at very low frequencies that are below the error rate of standard next-generation Illumina sequencing assays. Therefore, we used a powerful sequencing approach, Duplex Sequencing, that permits accurate detection of mtDNA variants at frequencies as low as one mutation in ∼10^8^ bp (37–39). Using this ultra-sensitive, mtDNA-targeted sequencing approach, we were able to detect more heteroplasmic mtDNA mutations at lower frequencies than ever before reported in *C. elegans*, and also investigated the mutational rates, spectrum, context, and genomic sites of mutagenesis after a long-term mutation accumulation experiment.

To test the effects of chemical stress and the role of mitophagy in the origin and transmission of mtDNA mutagenesis in *C. elegans*, we performed mutation accumulation experiments in wild-type and two mitophagy mutant strains, *pink-1* and *dct-1*, under various environmental conditions. All independent lineages (replicates) for each of the three strains were bottlenecked for 50 generations in control conditions or with chronic exposure for 50 generations to the heavy metal pollutant, cadmium (Cd, in the form of cadmium chloride, CdCl_2_) or the mycotoxin aflatoxin B_1_ (AfB_1_), both known nuclear mutagens and human carcinogens that have two different mechanisms of DNA damage. Cd inhibits many DNA repair enzymes and interferes with antioxidant enzymes, resulting in increased levels of ROS that can damage mtDNA (40,41). AfB_1_ metabolites form bulky DNA adducts, which can inhibit replication and result in somatic mutations in the nuclear genome (42). The accumulation of AfB_1_ in mitochondria, in addition to lack of nucleotide excision repair (NER), results in higher levels and greater persistence of mtDNA lesions compared to nuclear DNA damage, as we and others have previously measured (43–45). Therefore, we used these two toxicants as models to investigate how two different mechanisms of mtDNA damage may affect mtDNA mutational processes.

Duplex Sequencing of wild-type *C. elegans* mtDNA revealed a strong signature of oxidative damage. Exposure to a level of CdCl_2_ that is comparable to human blood levels and resulted in high levels of mtDNA damage, and a level of AfB_1_ that caused mtDNA damage and a significant increase in nuclear DNA mutation rate, did not increase the frequency of mtDNA single nucleotide mutations (SNMs) in wild-type *C. elegans*. Surprisingly, inhibiting mitophagy did not result in an increase in mtDNA mutations in control conditions or after exposure to CdCl_2_ or AfB_1,_ despite declines in fitness compared to wild-type *C. elegans*. These results suggest that the mitochondrial genome harbors robust mechanisms of avoiding chemical-induced mtDNA mutagenesis that may be independent of mitophagy.

## MATERIALS AND METHODS

### *C. elegans* strains and maintenance

This work used the *C. elegans* wild-type Bristol N2 strain and two mitophagy deficient strains, *dct-1* and *pink-1*. The *dct-1*(tm376) mutant harbors a 912 bp deletion in the promoter region of *dct-1* (DAF-16/FOXOControlled, germline Tumour affecting-1, putative orthologue to mammalian mitophagy receptor, BNIP3) and has been characterized previously (46). The *pink-1*(1779) mutant harbors a 350 bp deletion in *pink-1* (PTEN-induced kinase 1) and exhibits altered mitochondrial morphology (22). These deletion strains were acquired from the National Bioresource Project (Tokyo, Japan), genotyped, and backcrossed into N2 six times (*dct-1*) and eight times (*pink-1*) prior to any experiments. All *C. elegans* were maintained following standard procedures (47). We replaced the potassium phosphate in traditional nematode growth medium (NGM plates) with KCl (‘K-agar plates’) in order to prevent buffering which reduces the bioavailability of CdCl_2_ (48,49). All strains were maintained at 20°C on OP50 *Escherichia coli*.

### CdCl2 and AfB1 treatment

Stocks of CdCl_2_ and AfB_1_ (Sigma-Aldrich, St. Louis, MO) were made in ddH_2_O and DMSO vehicles, respectively, and stored at 4°C. Treatment plates were always made fresh: OP50 was spiked with either CdCl_2_ and AfB_1_ immediately prior to seeding, and plates were prepared two days prior to experiments. Reported concentrations are the concentrations in the spiked OP50. 100 μl of OP50 was seeded on 6cm plates (containing 8 ml of K-Agar) and 300 μl of OP50 on 10cm plates (containing 20 ml K-agar).

### Mitochondrial morphology

To assess effects of exposure on mitochondrial morphology, wild-type *C. elegans* harboring an extrachromosomal array P*myo-3*::GFP; which expresses GFP in the mitochondrial matrix of body wall muscle cells, were exposed to 10 μM and 50 μM of CdCl_2_ and 2 μM and 10 μM AfB_1_. Images were taken on a Keyence BZ-X700, and analyzed in ImageJ using the Blinder software (50). Blinded images were analyzed qualitatively by an established classification system as previously described (51), with Class 1 indicating highly networked, fused mitochondrial morphology, and Class 5 indicating extremely fragmented mitochondrial morphology. Two experimental replicates were conducted, and the total number of individuals analyzed is displayed above each stacked bar plot in Supplementary Figure S5. Statistical differences in the distribution of classes of mitochondrial morphology were determined by Fisher's exact test followed by Bonferroni correction for multiple comparisons.

### Mitochondrial respiration

Age-synchronized L1 *C. elegans* were transferred to plates seeded with OP50 containing 50 μM and 100 μM CdCl_2_, or 10 μM and 25 μM AfB_1_. Approximately 48 h later, L4 *C. elegans* were washed off the plates and respiration parameters were quantified using the Seahorse Extracellular Flux Bioanalyzer, as previously described (52). The number of individual nematodes per well was counted, such that the Oxygen Consumption Rate (OCR) measurements were normalized per worm. The mean of technical replicates per plate was then determined. Two to six independent experiments were conducted per strain per treatment. Two-way ANOVAs were run for each chemical in order to compare Cd to control and AfB_1_to control across three strains.

### Life history trait analyses

Growth was measured in three independent experiments in the ancestral (G0) wild-type, *dct-1* and *pink-1* strains on control and treatment plates. About 300 age-synchronized L1s were plated on each 10cm plate with the following conditions: control, 50, 200, 1000 μM CdCl_2_, and 10, 50 and 200 μM AfB_1_. After 48 h, L4 *C. elegans* were washed off the treatment plates and transferred to unseeded plates. Images of each plate were then captured with a Keyence BZ-X700 using brightfield, and analyzed in ImageJ with the Fiji plug-in WormSizer (53). Length data were normalized to the mean control length of each strain for each biological replicate. Box and whisker plots of length distributions were plotted in R and the means of biological replicates were used for statistical analysis (2-way ANOVA, Tukey's HSD post-hoc analysis). Total brood size was also determined in all three strains on control, 50 μM CdCl_2_ and 10μM AfB_1_ plates (*N* = 9–10 individuals per strain per treatment). On day 1 of adulthood, each individual worm was transferred to a new 6cm plate with the respective treatment until reproduction ceased. Plates containing offspring were stored at 20°C for 2 days, and then counted.

Population growth rate was measured on all MA lines at G0 and G50 to measure the effects of mutation accumulation on fitness. Population growth rate was determined by an eating race experiment as originally described by Hodgkin and Barnes (54). Each plate was monitored hourly up to the time when all bacteria had been consumed and the population dispersed. As an additional fitness measurement, total reproduction and lifespan were also determined on a subset of 20 MA lines per strain per treatment at G50. The 20 MA lines were determined with a random number generator. Of these 20 sublines, three L4 individuals (G50 sisters) were picked onto individual control plates. Founders were transferred to a new plate daily until cessation of egg-laying, and total brood was counted as described above. Biological replicates were then averaged per MA line. Individuals that were sterile were included in our analysis. Lifespan was determined by scoring individuals every day until they stopped responding to touch. Reproduction and lifespan results were censored if the animal was lost, dehydrated, or bagged. This only occurred in four of the 180 MA lines.

### mtDNA copy number and damage

OP50 was spiked with either CdCl_2_ or AfB_1_, such that the final concentrations of chemicals in the bacterial lawn were 50 μM of CdCl_2_ and 10 μM of AfB_1_. Plates were seeded with 100 μl of each treatment, and control, and allowed to dry for 2 days. 20–30 gravid adults of each strain, wild-type, *pink-1* and *dct-1*, were then picked onto each treatment plate. Adults were left on each plate for a 2-h egg-lay, after which all of the adults were removed from the plate. After 56 h, synchronized L4 *C. elegans* were then analyzed for mtDNA CN and damage, as described (55). Two to three biological replicates of six individual L4 *C. elegans* per strain per treatment were picked into 90 μl of lysis buffer, placed at −80°C for at least overnight, then lysed at 65°C for 1 h, followed by 95°C for 15 min to inactivate Proteinase K. Long-amplicon PCR was then performed (55), with one modification that the final reaction volume was halved (25 μl instead of 50 μl). mtDNA CN was measured using a plasmid-based standard curve and real-time PCR as described (56). mtDNA CN was normalized to the number of L4 individuals per sample (six) because nematodes have an invariant number of somatic cells (∼1000 cells), and thus invariant nuclear genome copies at this life stage. Long-amplicon PCR products were normalized to mtDNA copy number and mtDNA lesions were calculated relative to the control within each strain. Each biological sample was amplified in duplicate or triplicate. This experiment was performed twice. A two-way ANOVA followed by Tukey's HSD post-hoc analysis was used to determine statistical differences in mtDNA lesions between strains and treatment. Results of mtDNA lesions following exposure to additional concentrations of CdCl_2_ and AfB_1_ that were not used in the mutation accumulation line experiment are included in Supplementary Materials (Supplementary Figure S1). The effects of exposure on mtDNA CN are described in Supplementary Figure S2.

### Mutation accumulation lines

Our criteria to determine a single concentration of CdCl_2_ and AfB_1_ to use for mutagenesis experiments were: (i) the concentration caused significant mtDNA damage, but (ii) did not have significant effects on organismal fitness (growth, fecundity and mitochondrial toxicity). Once we determined a concentration for each exposure, we performed a mutation accumulation line experiment in wild-type, *pink-1* and *dct-1 C. elegans*, in control conditions, 50 μM CdCl_2_ or 10 μM AfB_1_ (Figure 2). One random young adult individual of each strain was isolated (G0 founder) and allowed to reproduce. Once the offspring reached gravid adulthood, about 20 individuals were picked onto a plate. After a 2-h egg lay, the adults were picked off the plate. When these offspring reached L4, one individual (G1 ‘subline’ with shared lineage from the founder) was randomly picked to an individual plate to begin the mutation accumulation experiment. The MA experiment began with 50 individual G1 sublines per strain per treatment. All lines were maintained at 20°C. Every four days, one individual L4 was randomly picked and transferred to a new plate to propagate the next generation. This population bottlenecking was conducted for 50 generations per strain per treatment per subline, resulting in a total of 2500 generations of mutation accumulation per strain per treatment. The previous generation was always maintained at 15°C, such that if an individual was sterile or dead, a back-up ‘sister’ individual could be transferred to a new plate to continue the MA line. As in previous *C. elegans* MA experiments, if three attempts were not viable, the line was then considered extinct (57). At the end of the experiment, the average percentage of extinctions per strain per treatment was 4.5% (Supplementary Figure S3). All 450 MA lines were cryopreserved every 5 generations for 50 generations.

### DNA isolation, duplex sequencing library preparation and sequencing of mtDNA

After 50 generations of mutation accumulation per MA line, an individual L4 from each MA line was transferred onto a control 6-cm plate. As has been previously described in other MA experiments, as soon as all of the OP50 had been consumed and the population was composed largely of synchronized L1s, the *C. elegans* were immediately washed off of the plate with ddH_2_O, transferred to a 1.7 ml Eppendorf tube, pelleted, and flash frozen in liquid nitrogen (34–36). Total DNA was isolated with the DNeasy Blood & Tissue kit (Qiagen, Germany) following the manufacturer's instructions. DNA quantity and purity was analyzed using a Thermo Scientific™ NanoDrop™ One Microvolume UV-Vis Spectrophotometer (Thermo Fisher Scientific) and Qubit 2.0 (Perkin Elmer Victor x2).

Illumina Sequencing libraries were prepared following the original Duplex Sequencing (DS) protocol with slight modifications (58,59). An input of 50 ng of total DNA was used for library preparation. Total DNA was sheared (Covaris E210), followed by preparation and repair of DNA fragments using the NEBNext Ultra II Library prep kit, in which 2 μl of 15μM of the unique DS adapters were added directly to the Ultra II Ligation Master Mix, according to manufacturer's instructions (New England Biolabs). Relative mtDNA copy number of the post-adapter ligated sample was then determined via RT-qPCR (Applied Biosystems StepOnePlus). This critical step determined the amount of post-ligated DNA to use as input for the pre-capture indexing PCR to optimize the number of Duplex Consensus Sequences formed for each Unique Molecular Identifier (59). The optimal family size was determined by earlier sequencing of a *C. elegans* wild-type sample compared to a known reference standard in order to determine volume of post-adapter-ligated for pre-capture indexing PCR. On average, 0.338 μl of template was required (range of 0.164–0.632). After the pre-capture indexing-PCR, the entire product was lyophilized with the addition of uniquely designed blocking oligos. An enrichment of the *C. elegans* mitochondrial genome was then performed following the IDT xGen Hybridization Capture of DNA libraries for NGS target enrichment protocol with a custom designed Discovery Pool probe panel that covered 13 091 bp of the 13 991 bp *C. elegans* mitochondrial genome, capturing 93.5% of the genome. The AT-rich, highly repetitive non-coding region of the genome was excluded in order to minimize sequencing fragments that would not accurately map to the reference genome. Post-capture PCR and clean-up were then performed using according to manufacturer's instructions. Libraries were sequenced on an Illumina NovaSeq 6000 platform to obtain 150 bp paired-end reads (20 million per library). The DS adapter sequences, qPCR and Illumina primer sequences, blocking oligo sequences, and the *C. elegans* mitochondrial genome custom probe panel oligonucleotide sequences are provided in the Supplementary Data File.

### Bioinformatics processing and analysis of mitochondrial genome sequences

Sequencing data were processed on the Duke Computer Cluster using the custom Duplex-Seq Pipeline (v1.1.4) workflow developed by the Kennedy Lab (github.com/Kennedy-Lab-UW/Duplex-Seq-Pipeline) and described in detail in Supplementary Figure 3 of Kennedy *et al.* (58) and again in Sanchez-Contreras *et al.* (11). Only reads mapping to the *C. elegans* mitochondrial reference genome were analyzed, and only single nucleotide mutations were analyzed, while insertions and deletions were ignored. Possible artifacts were removed by 12-bp end clipping at the beginning of each read. A minimum of three reads were required to call a variant, with a minimum heteroplasmy cutoff of 70% per Duplex Consensus Sequence (DCS) compared to the reference genome. Other input parameters are included in the Configuration File (Supplementary Data File). A summary output file of mutations, sequencing depth, and genome coverage per library is included (Supplementary Data File). Sequencing data is uploaded and can be accessed at SRA SRP350474 (PRJNA787252). We detected three polymorphisms that were completely fixed in our *C. elegans* strains compared to the reference genome (NC_001328.1), two of which have been previously detected (36). These polymorphisms were not included in our analysis and are listed in Supplementary Table S1.

### Mutation frequency and spectrum analysis

Each mutation was only called once at each genomic position. Overall mutation frequency was calculated for each library with the following equation: [total number of unique mutations]/[total number of error-corrected nucleotides sequenced]. For mutational signature calculations, the total number of each type of the six possible transition and transversion mutations was divided by the coverage of the reference nucleotides sequenced (i.e.: [total # C:G → A:T mutations]/[total number of cytosines + guanines sequenced]). Mean mutation frequencies were calculated for each strain and treatment, followed by parametric statistical analysis and appropriate multiple comparisons corrections, as described in Results.

### Trinucleotide context mutational signature

Trinucleotide context mutations were calculated using the Bioconductor Package *MutationalPatterns* (v1.1.0) (60) after forging a *C. elegans* mitochondrial reference genome (*BSgenome* v1.58.0). The number of mutations in each of the 96 possible trinucleotide contexts were determined in reference to pyrimidines C and T, hence six possible mutation types instead of 12. The number of each trinucleotide context mutation was normalized to the average number of wild-type control mutations in each context. A Welch two-sample *t*-test was performed on a specific mutation only after a significant ANOVA was determined.

### Synonymous and nonsynonymous mutation analysis

The dNdS ratio was calculated in order to determine if mtDNA evolution departed from neutrality in our MA experimental design. We used the *R* package *dNdScv*, a statistical modeling approach that allows for normalization to depth of coverage as a covariate, and incorporation of the invertebrate mitochondrial genetic code (translation Table 5) (61).

### Whole genome sequencing, processing, and *de novo* variant calling for nuclear DNA mutagenesis

We conducted whole genome sequencing of four control, four CdCl_2_, and four AfB_1_ wild-type MA samples to estimate nuclear DNA SNM rates. Sequencing libraries were created by the Duke University Genomics and Computational Biology Core from the same DNA templates that were used for Duplex Sequencing of the mitochondrial genome. Libraries were sequenced on an Illumina NovaSeq 6000 platform to obtain 150 bp PE reads.

Data quality control for the NGS raw fastq files was performed using the FastQC v0.11.8 to ensure no fundamental errors occurred during sequencing steps or library preparation of the samples. Reads were processed by Trimmomatic v0.39 to remove the 3’ end adapter sequences and trim low-quality sequences. Subsequently, sequence alignment of the processed reads was performed by mapping to the WBcel235 *C. elegans* Bristol N2 strain reference genome utilizing the BWA-MEM v0.7.17 aligner. SAMtools v1.14 flagstat was used to compute the number and percent of reads that mapped to the genome. Depth and breadth of coverage was computed using BEDTools v2.29.2. Post-alignment processing for the removal of read duplicates, indel realignment, and base quality score recalibration was completed using Picard v2.18.16 MarkDuplicates and the GATK v4.1.9.0 tools InDelRealigner and BaseRecalibrator. These post-alignment steps ensured high-quality aligned reads remained, which are necessary for the accurate detection of genomic variants including SNVs and CNVs. Germline variants were called using strelka v2.9.2 and population level SNPs and ancestral variants shared across all MA lines were filtered out. All variants were annotated with gene names, predicted function, population frequencies, and other variant annotations using Ensembl Variant Effect Predictor. Remaining *de novo* variants were filtered for depth, quality and 100% variant allele frequency.

### Statistical analysis

All statistical analysis and data visualization were conducted in RStudio (v1.1.463; *R* v4.0.3). Values are conveyed as means and standard error, unless otherwise indicated. We performed Welch Two Sample T-test to determine significance between two groups, and ANOVA (one way if more than two groups and two way if multiple factors, as indicated in further detail in the figure legends) followed by Tukey's HSD to correct for multiple comparisons. Fisher's exact test was performed to determine variation in the proportion of each type of mutation (Supplementary Figure S4).

## RESULTS

### CdCl_2_ and AfB_1_ exposure causes mtDNA damage in wild-type *C. elegans*

**Figure 1.**
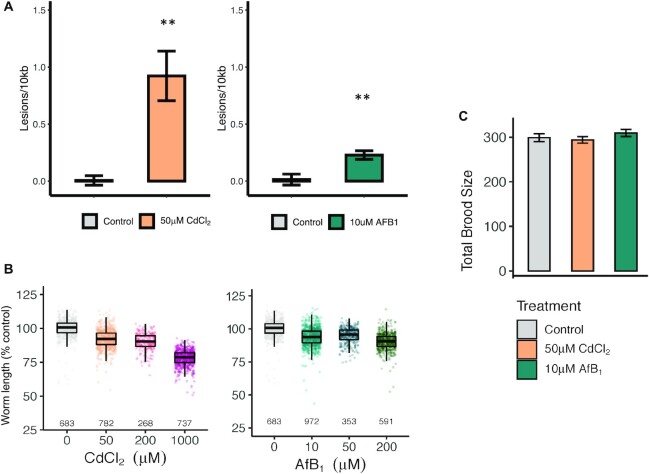
50μM CdCl_2_ and 10 μM AfB_1_ exposure induces mtDNA lesions in wild-type *C. elegans*, but has no effect on growth or reproduction. (**A**) mtDNA damage from pools of six individual age-synchronized L4 *C. elegans* was quantified after chronic exposure to control (*N* = 8), 50 μM CdCl_2_ (*N* = 8) or 10 μM AfB_1_ (*N* = 4) (** *P*< 0.01, one-way ANOVA). (**B**) A dose–response was conducted to measure effects on growth after 48 h of exposure (L1–L4). Each individual worm length was first normalized to the mean control length for each experiment. Dots represent technical replicates (individual nematodes, total *N* displayed) and the boxplots display the median and upper and lower quartiles. The mean normalized length was then calculated within each experimental replicate. There was no effect of growth after exposure to 50 μM or 200 μM CdCl_2_, but we observed 22% growth inhibition at 1000 μM CdCl_2_ compared to control (*P* = 0.15, *P*= 0.2, *P*= 0.009, respectively; one-way ANOVA, Tukey HSD). We did not observe growth inhibition after 10 μM or 50 μM AfB_1_ compared to control, but did observe 11% growth inhibition at 200 μM AfB_1_, though trending (*P*= 0.27, *P*= 0.59, *P*= 0.09, respectively; one-way ANOVA, Tukey HSD). (**C**) There was no effect of either 50 μM CdCl_2_ or 10 μM AfB_1_ on total brood size compared to control (*N* = 9–10, *P*= 0.9, *P*= 0.63, respectively; one-way ANOVA, Tukey HSD). Error bars indicate standard error (* *P*< 0.05; ** *P*< 0.01).

We have previously shown that exposure to CdCl_2_ and AfB_1_ results in significantly higher levels of mtDNA damage than nuclear DNA damage in wild-type *C. elegans* (43). In order to determine a single dose of CdCl_2_ and AfB_1_ to use for mutation accumulation lines and sequencing where there was detectable mtDNA damage but no evident effect on fitness, we first conducted a dose response and quantified mtDNA damage (Supplementary Figure S1), followed by measuring growth, reproduction, mitochondrial morphology, and mitochondrial respiration. Exposure to 50 μM CdCl_2_ induced 0.92 lesions/10 kb, and 10 μM AfB_1_ induced 0.25 lesions/10 kb relative to control in wild-type *C. elegans* (*P* < 0.01, Figure 1A). These concentrations of CdCl_2_ and AfB_1_ did not result in statistically significant growth delay (8.3% and 6.3% growth inhibition, respectively, Figure 1B) or fecundity (Figure 1C). We also observed little effect on mitochondrial morphology (Supplementary Figure S5) and no decrease in mitochondrial function (Supplementary Figure S6). Therefore, these concentrations were used for the mutation accumulation experiments to avoid any effects on fitness that may cause selective pressure within MA lines and therefore compromise mutation rate calculations.

### Wild-type *C. elegans* exhibit resistance to mtDNA damage-induced single nucleotide mutations

**Figure 2.**
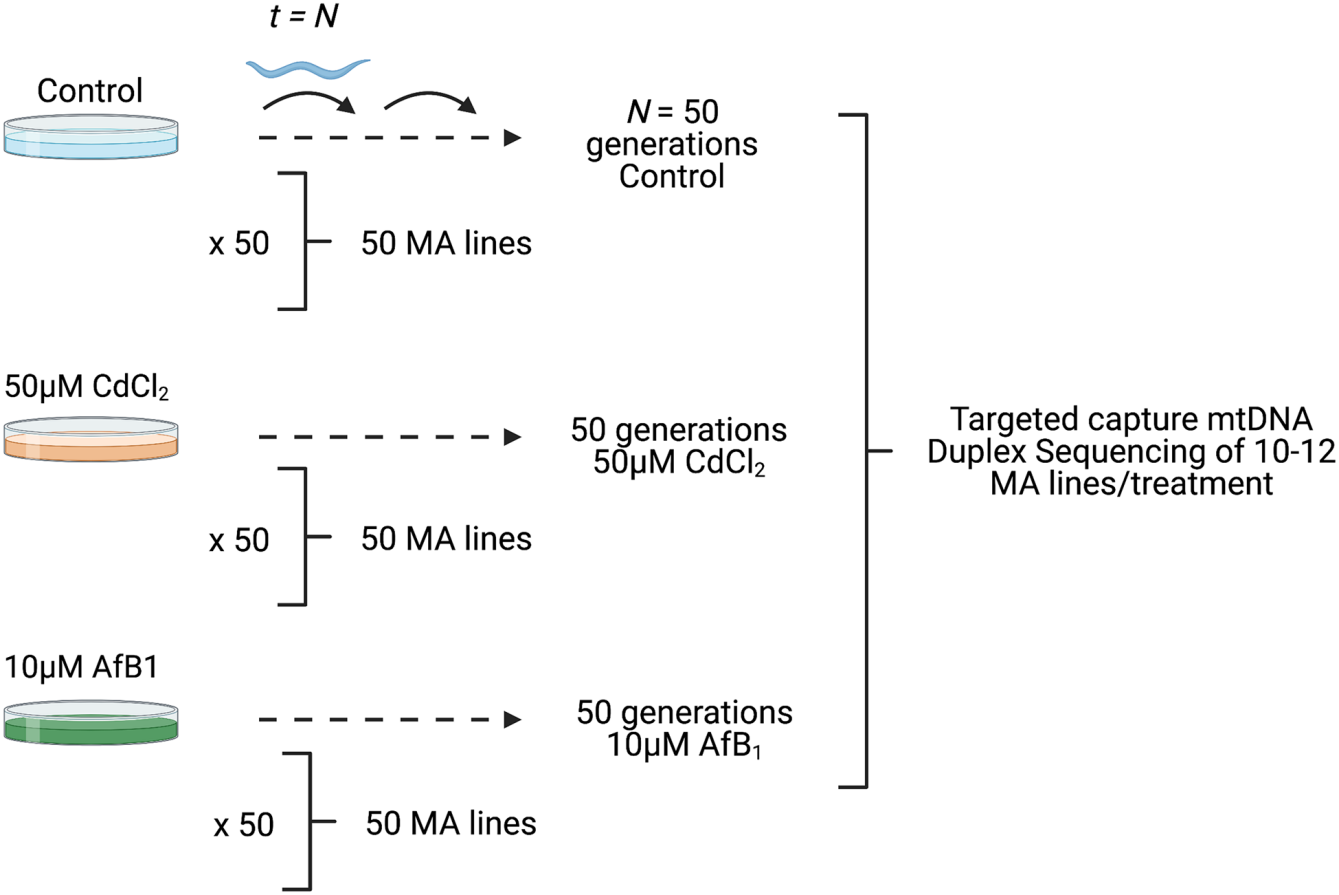
Schematic of mutation accumulation line experimental design. Offspring of a single founding ancestor (G0) were isolated onto individual plates. Every *t* = 4 days, a single L4 nematode was randomly selected and transferred to a new plate. This was repeated every generation (G) for 50 generations. We conducted MA lines on control plates, and plates that contained OP50 that was spiked with a final concentration of 50 μM CdCl_2_ and 10 μM AfB_1_. We conducted MA line experiments in wild-type *C. elegans* and two mitophagy mutant strains, *dct-1* and *pink-1*. 50 replicate ‘sublines’ were passaged for each strain*treatment. MA lines were randomly selected after 50 generations per subline (9–14 MA lines/strain/treatment) for life history analysis and targeted mtDNA Duplex-Sequencing. Image created with BioRender.com.

**Figure 3.**
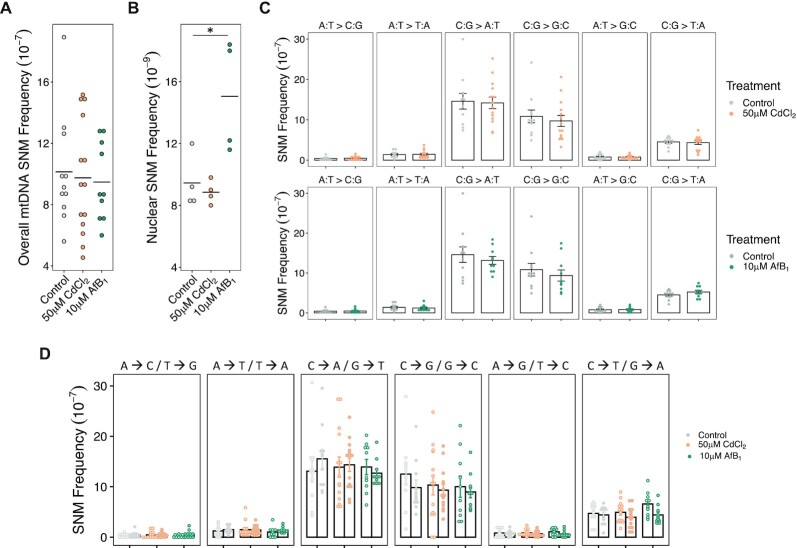
The mtDNA single nucleotide mutation signature in wild-type *C. elegans* is consistent with oxidative damage and demonstrates resistance to point mutations caused by CdCl_2_ and AfB_1_ mtDNA lesions. (**A**) Overall mtDNA SNM frequency was determined by Duplex Sequencing after 50 generations of mutation accumulation per subline in wild-type *C. elegans* in control conditions (gray dots; *N* = 11), as well as after 50 generations per subline of exposure to 50 μM CdCl_2_ (gold dots; *N* = 14) and 10 μM AfB_1_ (green dots, *N* = 10). There was no effect of either 50 μM CdCl_2_ or 10 μM AfB_1_ on overall mtDNA mutation frequency compared to control (*P*= 0.96, *P =*0.90, respectively); one-way ANOVA, Tukey HSD). Horizontal lines indicate mean values. (**B**) Overall nuclear SNM rates of a subset of MA lines in each treatment (*N* = 4 per treatment). There was no effect of 50 μM CdCl_2_ on overall SNM rate, but there was a 1.6-fold increase in overall SNM rate after exposure to 10 μM AfB_1_ (*P* = 0.01; ANOVA). Horizontal lines indicate mean rates. (**C**) mtDNA mutation spectrum of control, CdCl_2_ and AfB_1_-treated MA lines. Each dot represents a single MA line. The wild-type *C. elegans* mtDNA mutational signature was dominated by C:G → A:T and C:G → G:C transversion mutations, and there was no effect of Cd or AfB_1_ exposure on mtDNA mutational signature (Welch two-sample *t*-test). Error bars represent standard error of the mean. (**D**) mtDNA lesions may accumulate disproportionately on mtDNA strands, resulting in mtDNA mutation strand bias. We observed a trend towards an increase in C → T over G → A mutations after exposure to AfB_1_ compared to control (*P* = 0.08; two-way ANOVA, Tukey HSD). Open circles indicate one strand (i.e. A → C) and filled circles indicate the other strand (i.e. T → G).

The strong increase in mtDNA damage after CdCl_2_ and AfB_1_ suggested that mutations should be correspondingly increased. To test this hypothesis, we conducted Duplex Sequencing after 50 generations of mutation accumulation to determine the frequency and spectrum of mtDNA single nucleotide mutations on wild-type *C. elegans* in control and genotoxicant-treated conditions (Figure 2). We sequenced 11 wild-type MA lines and identified a total of 760 SNMs (an average of 70 mutations/MA line) under control conditions. The overall mtDNA mutation frequency of wild-type *C. elegans* was 10.1 × 10^–7^ SNMs/bp. Despite significant mtDNA lesions (Figure 1A), there was no effect of CdCl_2_ (14 MA lines, 874 total SNMs) or AfB_1_ (10 MA lines, 636 total SNMs) on the overall mtDNA SNM frequency in wild-type *C. elegans* (9.75 × 10^–7^ and 9.47 × 10^–7^, respectively) (Figure 3A, Supplementary Table S2). WGS was conducted in a random subset of four of the wild-type MA lines per condition in order to investigate the potential effects of CdCl_2_ and AfB_1_ on nuclear genome mutagenesis. We detected 189, 177 and 301 single nucleotide mutations in control, CdCl_2_ and AfB_1_-treated MA lines respectively after 50 total generations of mutation accumulation per treatment. We estimated a wild-type nuclear DNA mutation rate of 9.45 × 10^–9^ bp/site, which is very similar to previous estimates of nuclear DNA mutation rates in *C. elegans* mutation accumulation line experiments (62,63). We observed a 1.6-fold increase in the SNM rate in the AfB_1_ MA lines (15 × 10^–9^ bp/site, *P* = 0.016). There was no detectable effect of CdCl_2_ on overall nuclear DNA mutation rate (8.85 × 10^–9^ bp/site, *P* = 0.93) (Figure 3B).

The mtDNA mutation spectrum of *C. elegans* in all conditions was dominated by C:G → A:T and C:G → G:C transition mutations, and C:G → T:A mutations. There was no effect of CdCl_2_ or AfB_1_-induced mtDNA damage on mutation spectrum (Figure 3C; Welch two-sample *t*-test). Wild-type *C. elegans* exhibited no strand asymmetry in mtDNA mutation accumulation in control conditions (Figure 3D). There was no mutational strand asymmetry after exposure to CdCl_2_ (Figure 3D). We did observe a trend of a slightly higher frequency of C → T over G → A transversions after exposure to AfB_1_ compared to control conditions (*P* = 0.08; two-way ANOVA) (Figure 3D).

### Mitophagy deficiency impairs mtDNA damage removal but does not increase mtDNA mutation accumulation

**Figure 4.**
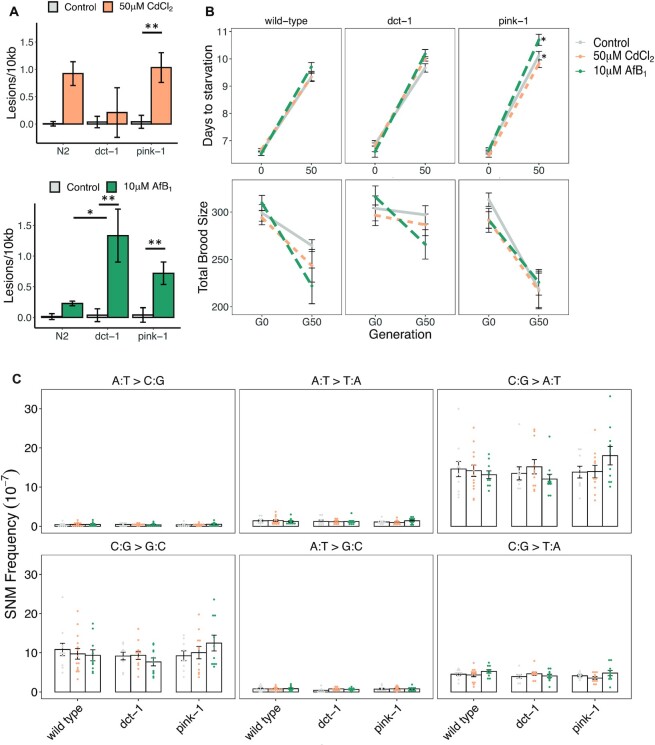
Mitophagy deficient mutants accumulate higher levels of mtDNA damage and exhibit greater fitness declines compared to wild-type, but exhibit no differences in mtDNA mutation frequencies after exposures. (**A**) mtDNA damage from pools of six individual age-synchronized L4 *C. elegans* after exposure to control (*N* = 8), 50 μM CdCl_2_ (*N* = 8) or 10 μM AfB_1_ (*N* = 8). *pink-1* mutants accumulated mtDNA damage after exposure to CdCl_2_, but not more than wild-type, while *dct-1* mutants did not accumulate mtDNA damage. Both mutants accumulated high levels of mtDNA damage after exposure to AfB_1,_ and *dct-1* accumulated significantly higher levels compared to wild-type exposed *C. elegans*. Error bars indicate standard error of the mean (* *P* < 0.05; ** *P*< 0.01; two-way ANOVA, Tukey HSD). (**B**) Population growth rate and total brood size as indicators of fitness in ancestors (G0) and after 50 generations of MA in all lines (G50). Each dot represents the mean value of days to starvation (until all of the food was eliminated and the population dispersed on the plate), with error bars as standard error of the mean (*N* = 3 for G0, *N* = 39- 48 for G50 MA lines). Lines indicate the rate of change in fitness from G0 to G50 (gray solid = control, gold small dash = 50 μM CdCl_2_, and green large dash = 10 μM AfB_1_) for wild-type, *dct-1*, and *pink-1* strains. Y-axis lower limit begins at Day 6. There was no effect of 50 μM CdCl_2_ or 10 μM AfB_1_ on population growth rate in any strain at G0. All MA lines had significantly slower population growth rate at G50 compared to G0 (two-way ANOVA, *P <*2.2e–16). There was no effect of either CdCl_2_ or AfB_1_ on wild-type MA lines at G50 (*P*= 1, *P*= 0.95, respectively; two-way ANOVA, Tukey HSD). There was no effect of either CdCl_2_ or 10 μM AfB_1_ on *dct-1* MA lines at G50 (*P*= 0.98, *P*= 0.6, respectively; two-way ANOVA, Tukey HSD). There was no effect of either CdCl_2_ or 10 μM AfB_1_ on *pink-1* MA lines at G50 (*P*= 1, *P*= 0.4, respectively; two-way ANOVA, Tukey HSD). Both control and 10 μM AfB_1_*pink-1* MA lines had a significantly greater decline in fitness compared to control and 10 μM AfB_1_ wild-type MA lines (*P*= 0.03, *P =* 0.0008, respectively; two-way ANOVA, Tukey HSD). At G50, three individual L4s per MA line were transferred onto an individual control plate for brood size experiments. Each individual was transferred every day until cessation of egg-laying. Reproduction was counted on the previous plate after 48 h. Each dot represents the mean value total brood size per MA line, with error bars indicating standard error of the mean (*N* = 9–10 for G0, *N* = 18–20 for G50 MA lines). Lines indicate the rate of change in fitness from G0 to G50 (gray solid = control, gold small dash = 50 μM CdCl_2_, and green large dash = 10 μM AfB_1_) for wild-type, *dct-1*, and *pink-1* strains. The y-axis lower limit begins at 190. All MA lines had significantly lower fecundity at G50 compared to G0 (*P =*2.596e–11; two-way ANOVA). However, there was no effect of either CdCl_2_ or 10 μM AfB_1_ on total brood size in wild-type MA lines (*P*= 1, *P =*0.7, respectively; two-way ANOVA, Tukey HSD). *pink-1* control MA lines had significantly smaller broods than *dct-1* control MA lines at G50 (*P*< 0.01; two-way ANOVA, Tukey HSD) and *pink-1* CdCl_2_ MA lines had significantly smaller broods than *dct-1* CdCl_2_ MA lines at G50 (*P*= 0.03), suggesting a decrease in fitness in *pink-1* MA lines compared to *dct-1* MA lines. (**C**) Mutation spectrum of control (gray), CdCl_2_ (gold), and AfB_1_ (green) treated MA lines in wild-type, *dct-1*, and *pink-1* strains. The mitophagy mutant *C. elegans* mtDNA mutational signatures were also dominated by C:G → A:T and C:G → G:C transversion mutations, and there was no effect of CdCl_2_ or AfB_1_ exposure on *dct-1* or *pink-1* mtDNA mutational signature (two-way ANOVA). Error bars indicate standard error of the mean.

Given the surprising finding that exposure to well-known mutagens did not result in increased mtDNA mutations over control, despite the increase in mtDNA damage and increased nuclear mutation rates after AfB_1_ exposure, we hypothesized that mitophagy may be working to remove the damaged mitochondria before having a chance to form mutations. To test this hypothesis, we conducted a similar MA exposure experiment in mitophagy deficient strains. We chose *dct-1* and *pink-1* deficient strains because *dct-1* and *pink-1* (BNIP-3 and PINK1 human homologs) are involved in two independent mitophagy pathways (64). BNIP3 is a receptor-mediated pathway that, when activated, directly interacts with LC3 to facilitate autophagosome formation and mitochondrial degradation (65). This pathway is activated in response to hypoxia and nutrient depravation. PINK1 mediated mitophagy is activated by loss of mitochondrial membrane potential, resulting in the stabilization and accumulation of PINK1 on the outer mitochondrial membrane. PINK1 phosphorylates ubiquitin, followed by recruitment of Parkin E3 ubiquitin ligase which then recruits autophagy adapters to deliver mitochondrial to lysosomes for degradation (66). In our experiments, mitophagy-deficient *dct-1* mutants did not accumulate significant mtDNA lesions compared to control after exposure to CdCl_2_ (0.21 lesions/10 kb), but did after exposure to AfB_1_ (1.33 lesions/10 kb, *P* < 0.01; two-way ANOVA, Tukey HSD) (Figure 4A). *pink-1* mutants accumulated significantly higher levels of mtDNA damage after exposure to CdCl_2_ and AfB_1_ respectively compared to control (1.03 and 0.72 lesions/10 kb, *P* < 0.01; two-way ANOVA, Tukey HSD) (Figure 4A). Neither *dct-1* or *pink-1* mutants accumulated higher levels of mtDNA damage after exposure to CdCl_2_ compared to wild-type (two-way ANOVA). *dct-1* mutants did accumulate significantly higher levels of mtDNA damage compared to wild-type after exposure to AfB_1_ (*P* < 0.05; two-way ANOVA, Tukey HSD) (Figure 4A). *pink-1* mutants may accumulate higher levels of mtDNA damage compared to wild-type after AfB_1_ exposure; this trended towards, but did not reach, significance (*P =*0.08; two-way ANOVA, Tukey HSD) (Figure 4A).

Spontaneous mutagenesis due to mutation accumulation results in negative fitness consequences (67,68). Therefore, we assayed population growth rate and reproduction in all of the mutation accumulation lines after 50 generations of bottlenecks per strain per treatment per subline (Figure 4B). We quantified the time to starvation as the number of days that a population derived from a single L4 consumed all of the food source on the plate (54), as well as brood size (number of progeny per individual). There was no effect of 50 μM CdCl_2_ or 10μM AfB_1_ on population growth rate in any strain at G0 (Figure 4B). As expected, all MA lines had significantly slower population growth rate at G50 compared to G0 (two-way ANOVA, *P <* 2.2e-16). There was no effect of either CdCl_2_ or AfB_1_ on wild-type MA lines at G50 (*P* = 1, *P* = 0.95, respectively; two-way ANOVA, Tukey HSD), on *dct-1* MA lines at G50 (*P* = 0.98, *P* = 0.6, respectively; two-way ANOVA, Tukey HSD) or on *pink-1* MA lines at G50 (*P* = 1, *P* = 0.4, respectively; two-way ANOVA, Tukey HSD). However, both control and 10μM AfB_1_*pink-1* MA lines had a significantly greater decline in fitness compared to control and 10μM AfB_1_ wild-type MA lines, with about a 10% decline in population growth rate (*P* = 0.03, *P =* 0.0008, respectively; two-way ANOVA, Tukey HSD). We quantified total brood size per individual at G50 in a subset of MA lines (*N* = 20 per strain per treatment). All MA lines had significantly lower fecundity at G50 compared to G0 (*P =*2.596e–11; two-way ANOVA). However, there was no effect of either CdCl_2_ or AfB_1_ on total brood size in wild-type MA lines (*P* = 1, *P =*0.7, respectively; two-way ANOVA, Tukey HSD). *pink-1* control MA lines had significantly smaller broods than *dct-1* control MA lines at G50 (*P* < 0.01; two-way ANOVA, Tukey HSD) and *pink-1* CdCl_2_ MA lines had significantly smaller broods than *dct-1* CdCl_2_ MA lines at G50 (*P* = 0.03). The lower fecundity and slower population growth rate suggests a greater decrease in fitness in *pink-1* MA lines compared to wild-type and *dct-1* MA lines (Figure 4B).

In order to determine if the decline in fitness could be due to accumulation of mtDNA damage-induced mutagenesis in the mitophagy-deficient strains, we sequenced *dct-1* and *pink-1* MA lines after 50 generations of mutation accumulation in control, CdCl_2_, and AfB_1_-treated conditions. A total of 491, 727 and 574 total SNMs were detected after sequencing 11 control, 11 CdCl_2_ and 9 AfB_1_*dct-1* MA lines, respectively. A total of 530, 696 and 752 total SNMs were detected after sequencing 9 control, 11 CdCl_2_, and 10 AfB_1_*pink-1* MA lines, respectively. There was no effect of mitophagy deficiency or exposure on overall mtDNA mutation frequency (Supplementary Table S2, Supplementary Figure S7; two-way ANOVA, *P =*0.3). As in wild-type, the mutational spectra of *dct-1* and *pink-1* MA lines were also dominated by C:G → A:T and C:G → G:C transitions, and C:G → T:A transversions (Figure 4C). There was no effect of CdCl_2_ or AfB_1_ on the mutational spectrum in either *dct-1* or *pink-1* mutants (two-way ANOVAs).

### Trinucleotide context mutagenesis

**Figure 5.**
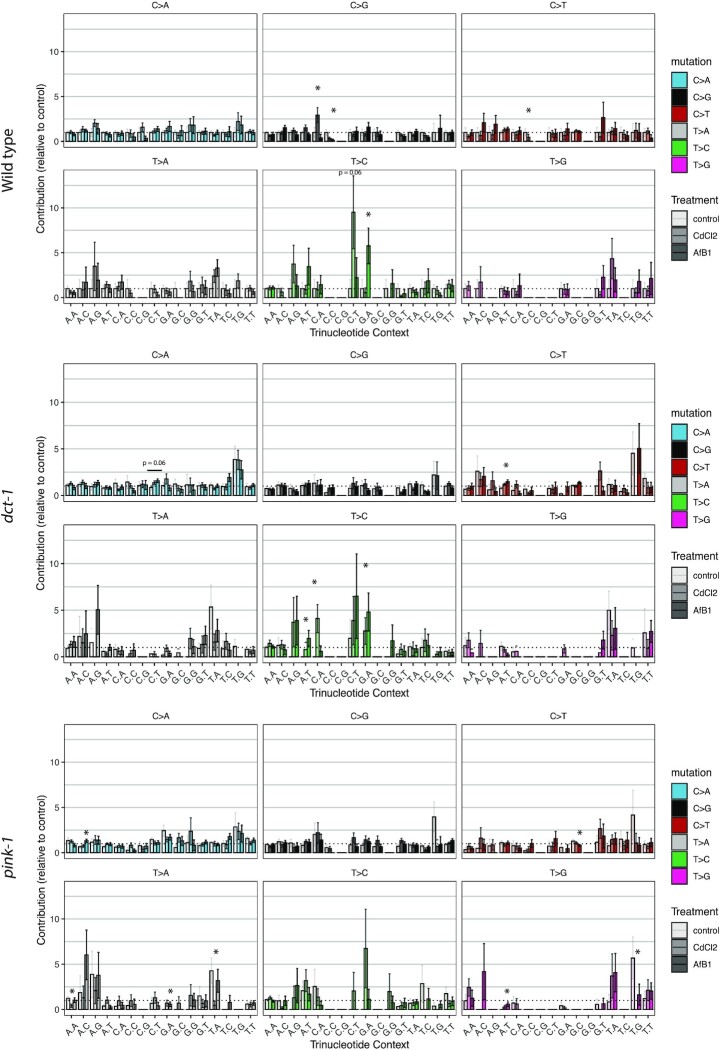
Trinucleotide context mutational signature of wild-type, *dct-1* and *pink-1 C. elegans* after exposure to CdCl_2_ or AfB_1_. Contributions of each of the 96 possible trinucleotide mutations were determined for each MA line, and then normalized to the mean wild-type control contribution (show as a dashed line) to determine effects relative to wild-type control. Bar graphs show the relative mean and standard error of each trinucleotide context mutation, with each mutation represented by a different color and control, CdCl_2_ and AfB_1_ treatment as increasing color hues. Potential effects of each exposure compared to control were determined within each mutation type (Welch two-sample *t*-test) only after a significant ANOVA was determined. * *P*< 0.05.

Mammalian mtDNA has been reported to have a distinctive mutational signature (69). Therefore, we analyzed the identity of the 5’ and 3’ neighboring nucleotides at each SNM to investigate possible enrichment of SNMs in specific sequence contexts to reveal mechanisms of mtDNA mutagenesis in *C. elegans*. Overall, the *C. elegans* mitochondrial genome does have a distinct trinucleotide mutational signature (Supplementary Figure S8). Specifically, C:G → T:A mutations occur in a G[C→T]C context, while C:G → A:T mutations occur mainly at A[C/G]A, X[C/G]T, T[C/G]A and T[C/G]T sites. In order to determine the effect of chemical-induced mtDNA damage on trinucleotide context mutagenesis, we normalized the relative contribution of each mutation to the mean wild-type control contribution (Figure 5). In wild-type exposed to CdCl_2_, there were more C → G mutations in a C[C/G]A context compared to control (*P* < 0.05; Welch two-sample *t*-test). There was also a trend of more T → C mutations in a C[T/A]T context compared to control (*P* = 0.06; one-way ANOVA, Welch two-sample *t*-test). In AfB_1_ MA lines, we observed fewer C → G and C → T mutations in a C[C/G]C context, and more T → C mutations in a C[T/A]A context compared to control (*P* < 0.05; one-way ANOVA, Welch two-sample *t*-test). In *dct-1* mutants, we observed more T → C mutations in CdCl_2_ MA lines in a C[T/A]A context compared to control (*P* < 0.05; one-way ANOVA, Welch two-sample *t*-test), and more C → T in a A[C/G]T context and T → C in A[T/A]T and G[T/A]A contexts after exposure to AfB_1_ compared to control (*P* < 0.05; one-way ANOVA, Welch two-sample *t*-test). In the *pink-1* mutants, we observed lower relative contributions of mutations at specific trinucleotide contexts compared to wild-type control, with the exception of more C → A mutations in a A[C/G]C context in AfB_1_ compared to *pink-1* control (*P* < 0.05; one-way ANOVA, Welch two-sample *t*-test).

We used the software *MutationalPatterns* to determine the cosine similarities of trinucleotide context mutational signatures between strain and treatment. As there were very few effects in the enrichment of trinucleotide mutations due to treatment as described above, it was unsurprising that the trinucleotide context mutational signatures were highly similar between strains and treatment (Figure 5 and Supplementary Figure S8). Indeed, the cosine similarity values across the three strains and three conditions were all close to 1, indicating high similarity (Supplementary Table S3). We next determined the similarity of these mutational signatures to known mutational processes as described in the Catalogue of Somatic Mutations in Cancer (COSMIC) database (http://cancer.sanger.ac.uk/cosmic/signatures) (Supplementary Table S4) (70). It is critical to note that the COSMIC signatures are derived from somatic nuclear mutagenesis from human cancer genomes, and *C. elegans* germline mitochondrial mutational processes are likely very different. Taking this into consideration, it was compelling that across all of our samples we detected high cosine similarity to the single base substitution (SBS) mutational signature that is associated with nucleotide excision repair deficiency (SBS24), which is absent in mitochondria. There was also high similarity with signatures associated with tobacco exposures (SBS4, SBS29). Tobacco products contains high levels of cadmium and smoke contains high levels of benzo[a]pyrene, which results in the formation of DNA adducts similar to those caused by AfB_1_. There also exists high similarity between signatures in which high levels of ROS is the proposed etiology (SBS18), in addition to defective base excision repair due to ROS-induced DNA damage (SBS36) and replication error across abasic sites (SBS13a). The similarities of *C. elegans* mtDNA signatures with these COSMIC signatures are likely due to the high C:G → A:T mutational bias in the *C. elegans* mitochondrial genome. Cosine similarity values are in Supplementary Table S4 and S5.

## DISCUSSION

Mutation accumulation experiments in *C. elegans* and other organisms have determined that chemical exposures contribute to mutagenesis in the nuclear genome (71,72). However, the role of chemicals in mtDNA mutagenesis is not well studied (73). mtDNA is resistant to single nucleotide mutations in somatic tissues after exposure to the polycyclic aromatic hydrocarbon benzo[a]pyrene and hydrogen peroxide (9,15), and in germ cells after exposure to hydrogen peroxide (74). Mitophagy is responsible for degrading organelles with damaged mtDNA (23) and purifying selection against highly deleterious deletion mutations in the germline (31,75). Indeed, placing a Pol γ mutator strain in a mitophagy-deficient background resulted in increased mtDNA mutation frequency in *C. elegans* (76,77). However, to our knowledge, empirical evidence for or against a role of mitophagy in ameliorating chemical-induced mtDNA damage, or mutations caused by damage in the germline, had not been reported. We found that *C. elegans* mtDNA is resistant to germline mutagenesis from exposure to known nuclear mutagens Cd and AfB_1_, despite accumulating high levels of mtDNA damage. Unexpectedly, we also found that accumulation of spontaneous single nucleotide germline mutations appears to be independent of mitophagy.

Previous *C. elegans* mutation accumulation line experiments have been instrumental in our understanding of mtDNA mutation rates (8,34). However, various intricacies of mitochondrial genomics in *C. elegans* and other species complicate the study of mtDNA mutagenesis (78). The mitochondrial genome exists in multiple copies per organelle and cell, meaning that multiple genomes can pass through the germline bottleneck (∼60 copies in *C. elegans*) (34), potentially allowing selection to still act on the organelle and cellular level even in a neutral mutation accumulation design (79). Because mtDNA is polyploid, the frequency of each *de novo* single nucleotide mutation is often lower than the limit of detection of traditional next-generation sequencing (error rate of 1 in 10^3^ bp). Furthermore, mutations that result from DNA damage are likely even more rare than spontaneous mutagenesis (80).

We sequenced 96 MA lines after about 50 generations of mutation accumulation each, resulting in a total of ∼4,800 generations of mutation accumulation. Highly sensitive Duplex Sequencing allowed us to detect a total of 6040 SNMs, which is about 250-fold higher than any previous *C. elegans* mitochondrial mutation accumulation line experiment to date. Our results were likely not biased by selection, as dN/dS ratios were ∼1 across all MA lines (Supplementary Table S6) (81,82). We observed a mutation spectrum similar to that reported in a recent Duplex Sequencing study of wild-type *C. elegans*, though roughly a 5-fold higher mutation frequency, which might be attributed to the higher number of generation bottlenecks in our experiment (50 generations of one individual versus three generations of passaging 10 individuals) or other differences in methodological and computational parameters (36). High frequencies of C:G → A:T and C:G → G:C transversion mutations support the conclusion that oxidative damage may drive mtDNA mutagenesis in *C. elegans*. The mechanism underlying this spectrum may relate to the apparent absence of some BER enzymes (83–85) or highly oxidizing intracellular environment under extreme drift (86). Mitochondrial redox state and oxidative mtDNA damage have not been measured in *C. elegans*, but could explain the high C:G → A:T mutation frequency in our MA experiment.

We observed a dominant G[C→T]C trinucleotide mutation across all strains and treatments (Supplemental Figure S6). This signature has been detected in various studies including aged human populations and has also been observed in some cancers (87). This suggests that either deoxycytidine deamination readily occurs in the *C. elegans* mitochondrial genome, or oxidized cytosines contribute to this mutational pattern. Cytosine deamination due to oxidative stress results in an excess of C → T over G → A mutations on the mtDNA heavy strand in many different organisms including *Drosophila* (9), mouse (88) and human mtDNA (89), and is likely the primary source of Pol γ error-induced mtDNA mutagenesis (11). However, our results did not reveal any strand asymmetry in mutation frequencies in wild-type *C. elegans* as would be predicted, particularly since oxidative damage is the prevailing model of mtDNA mutagenesis in *C. elegans* (36). Lack of strand-asymmetric mutagenesis could be because the mechanism of mtDNA replication in *C. elegans* may deviate from the single-strand displacement theory of other organisms (90), perhaps restricting exposure of damage-prone single-stranded mtDNA to oxidative stress and thus preventing strand bias. The potential compensatory mechanisms that have evolved to evade the consequences of high rates of oxidative damage-induced mutagenesis in *C. elegans* remains an intriguing area of study (91,92).

Mitochondria are a target of Cd (93), but the genotoxic and mutagenic effects of Cd on mtDNA are unknown. Cd is a ubiquitous heavy metal that is released from a range of sources, including mining, smelting, and combustion. The most significant sources of human exposure to Cd are contaminated food, smoking, e-waste sites, and products such as toys, jewelry, and plastics (94). Cd is a known carcinogen (95). It inhibits nuclear DNA repair mechanisms such as mismatch repair (40,96–98) and base excision repair (99), as well as zinc-dependent enzymes. Cd induces oxidative stress, likely via antioxidant depletion mechanisms (41). We found that exposure to 50 μM CdCl_2_ resulted in high levels of mtDNA damage across all genotypes, yet to our surprise, we observed no effect of genotoxicity on mtDNA SNM frequency or spectrum (Figures 1A, and 4A). There was almost no effect on the trinucleotide context mutational signature after exposure to CdCl_2_, except for an enrichment of a C → G transversion mutation in a C[N]A context compared to control in wild-type *C. elegans*. This may be indicative of oxidative damage, as 2,5-diamino-4H-imidazole-4-one (Iz) lesions contribute to C → G transversions in *E coli* (100). A recent study determined that human Pol γ is more prone to preferentially misincorporate a G opposite 2,2,4-triamino-5(2 *H*)-oxazolone, the common hydrolyzed product of Iz (101,102). We did not have the capability to identify the specific type of mtDNA lesions that are a result of Cd exposure, but the preponderance of this C → G transversion mutation could suggest that Cd causes a forms of oxidative damage, beyond 8-oxo-7,8-dihydro-2′-deoxyguanosine (8-oxodG) lesions which are likely repaired in the mitochondria, and thus contributes to C → A/G → T transversions. The mechanism by which oxidative damage and repair contributes to mtDNA mutational spectrum in *C. elegans* mtDNA mutagenesis is unclear, but *C. elegans* is able to repair at least some forms of oxidative mtDNA damage (85,103–106), despite the aforementioned absence of clear homologues of some BER enzymes.

Neither of the mitophagy mutant strains accumulated significantly higher levels of mtDNA damage after exposure to 50μM CdCl_2_ compared to exposed wild-type. Because CdCl_2_ by itself did not result in detectable mtDNA mutations, the lack of an increase in mutations in mitophagy mutants after CdCl_2_ cannot be interpreted to rule out mitophagy-mediated removal of CdCl_2_-induced mutations. *dct-1* mutants do have an enrichment of one T → C transition mutation in a C[N]A context in Cd conditions compared to control, but the process underlying this mutation is unknown. It is possible that the quantity of lesions formed at this concentration of Cd was not enough to contribute to mtDNA mutagenesis. However, the internal body burden of Cd in *C. elegans* after exposure to 50μM CdCl_2_ (Supplementary Table S7) is equivalent to levels detected in human blood (95), suggesting that this concentration is relevant to human exposures.

As mentioned, *C. elegans* can repair at least some oxidative mtDNA damage. To investigate the effect of irreparable mtDNA damage on mutagenesis, we used AfB_1_. AfB_1_ is a mycotoxin produced by the fungus *Aspergillus flavus* and is one of the leading causes of hepatocellular carcinoma worldwide. *A. flavus* grows on staple grains and legumes, and is metabolized when consumed to an epoxide that forms stable AfB_1_-N^7^-G adducts that cause G → T mutations in the nuclear genome, primarily in CGC trinucleotide contexts (42). The *C. elegans* and mammalian nuclear mutational spectra are similar (62,72). However, to our knowledge, no study has investigated effects on mtDNA mutagenesis, even though it has been known for decades that AfB_1_ preferentially attacks mtDNA over nuclear DNA (45).

Mitophagy is required to remove AfB_1_-induced lesions, yet we measured only very small effects of 10μM AfB_1_ exposure on mtDNA mutational spectrum in wild-type and mitophagy-deficient *C. elegans*. We did observe a trend towards an increase in C:G → A:T transition mutations in AfB_1_-exposed *pink-1* mutants compared to wild-type (1.4-fold greater mutation frequency, *P =* 0.07, two-way ANOVA, Tukey HSD). This suggests that *pink-1* may play a role in ameliorating mtDNA damage or mediating purifying selection against deleterious mutations. Over 50 generations of mutation accumulation, *pink-1* mutants had a greater fitness decline than wild-type and *dct-1* mutants. This is consistent with increased accumulation of deleterious mutations, but we cannot rule out mutations in the nuclear genome or nongenetic effects (109).

The trinucleotide context mutational signature in AfB_1_ MA lines did not reveal the clear signature that has been observed in nuclear genome studies. There is a significant but small enrichment of C → A mutations in a A[C/G]C context in AfB_1_ MA lines compared to control in *pink-1* mutants, which varies slightly from the known nuclear C[G]C context, suggesting a subtle but unique mechanism of aflatoxin damage-induced mutagenesis in the *C. elegans* mitochondrial genome. The unique AfB_1_ signature may result from the fact that damage removal processes are different for the two genomes; AfB_1_–induced damage in the nuclear genome is repaired by the enzyme NEIL1 as well as NER (107), but NER does not operate in the mitochondria and *C. elegans* does not appear to have a NEIL1 gene.

Mitophagy does regulate the transmission and accumulation of mtDNA mutations (31,108), yet our results suggest that this is perhaps limited to high-frequency, highly deleterious large deletion contexts. We found, for the first time, that constitutive mitophagy is responsible for removing mtDNA damage caused by AfB_1_ exposure (Figure 3A). A limitation of our work is that we did not measure mitophagy *per se* after exposure, though exposure to Cd and AfB_1_ induces mitophagy in other species (109–112). However, previous work in our lab has demonstrated that mitophagy is required to remove mtDNA damage caused by UVC exposure in *C. elegans*, but induction of mitophagy is not required (23,24,113). It is also possible that mtDNA damage exacerbates polymerase error-mediated accumulation of deletion mutations that we are unable to detect with Duplex Sequencing. Future studies should include technologies such as LostArc that can estimate frequencies of *de novo* deletions (114). Taken together, the current evidence suggests that *de novo* single nucleotide mutations present at low frequency do not have severe phenotypic consequences on organelle function and therefore evade targeted degradation via mitophagy.

Overall, our study suggests that in *C. elegans*, mitochondria harbor important quality control processes that are perhaps complementary or redundant to the two mitophagy pathways investigated in this study, resulting in a remarkable resistance to chemical-induced mutagenesis. A recent study in *Drosophila* found that unhealthy organelles failed to import nuclear-encoded factors essential for mtDNA replication and biogenesis. This resulted in the replication of wild-type mtDNA in healthy organelles, and not mutant mtDNA (115). Perhaps a ‘replication-competition’ model remains to be discovered in *C. elegans*. Direct degradation of damaged or mutant mtDNA may also play a protective role (116), though to our knowledge, *C. elegans* lacks a homolog for mitochondrial genome maintenance exonuclease (MGME-1) that degrades linear and damaged mtDNA at the mtDNA replisome. Some mechanisms that target paternal mitochondria and mtDNA for degradation in *C. elegans* are known, and an intriguing possibility is that similar pathways that target damaged or mutant mtDNA are present in the germline, which could suppress accumulation of deleterious mtDNA variants (117,118). On the organelle level, mitochondrial fusion and fission play a significant role in mediating mitochondrial quality control and are prerequisites for mitophagy (21,24). At 50μM CdCl_2_ and 10μM AfB_1_ exposure, we did not observe fragmentation of the *C. elegans* body wall muscle mitochondrial network in G0 wild-type *C. elegans* compared to control. We observed increased mitochondrial fusion at lower levels of Cd and AfB_1_exposure, with recovery at higher doses, which suggests that mitochondrial dynamics responded to Cd and AfB_1_-induced toxicity (Supplementary Figure S5). Future studies should investigate the role of mitochondrial dynamics in organelle and mtDNA turnover and biogenesis in the context of other chemical exposures. Lastly, another potential mechanism of ameliorating the removal of damaged mtDNA in the germline on the organismal level is germline apoptosis, which has previously been reported to increase after Cd and AfB_1_ exposure in *C. elegans*, though not at the concentrations that were used in our study (119,120).

The use of chemicals in society has resulted in over 86,000 chemicals of unknown toxicity in production (121), in addition to the hundreds of thousands of tons of pharmaceutical and industrial waste, increases in air pollution, and other sources of pollution that impact the health of hundreds of millions of people around the globe daily (122). Many chemicals can cause genome instability, which drives cancer development and other diseases; therefore it is necessary to better understand how low-level exposures to environmental agents can promote mutagenesis (123,124). mtDNA mutagenesis contributes to cancer and other diseases (69,125–128), yet is far less understood than nuclear DNA mutagenesis. Mitochondria are vital organelles that are highly susceptible to toxicity and genomic damage (17,129,130). MtDNA mutations accumulate with age, yet there remains conflicting evidence in the literature for the role of genotoxicant exposure in mtDNA mutagenesis (73). Surprisingly, we found that in *C. elegans*, after hundreds of generations of continuous chemical exposure, with hundreds of SNMs detected, and in the context of loss of two key mitophagy genes, mtDNA is resistant to damage-induced single nucleotide mutations. Future studies to investigate the effect of more chemicals in various genetic backgrounds may elucidate mechanisms in which mitochondria resist chemical-induced point mutations.

## DATA AVAILABILITY

The Duplex-Seq-Pipeline is written in Python and R, but has dependencies written in other languages. The Duplex-Seq-Pipeline software has been tested to run on Linux, Windows WSL1, Windows WSL2 and Apple OSX. The software can be obtained at https://github.com/Kennedy-Lab-UW/Duplex-Seq-Pipeline under the BSD license.

The data are available as raw reads under BioProject PRJNA787252.

## Supplementary Material

gkac666_Supplemental_Files

## References

[1] Johnston I.G. , Williams B.P. Evolutionary inference across eukaryotes identifies specific pressures favoring mitochondrial gene retention. Cell Syst. 2016; 2:101–111. 27135164 10.1016/j.cels.2016.01.013

[2] Bannwarth S. , Procaccio V., Lebre A.S., Jardel C., Chaussenot A., Hoarau C., Maoulida H., Charrier N., Gai X., Xie H.M. et al. Prevalence of rare mitochondrial DNA mutations in mitochondrial disorders. J. Med. Genet. 2013; 50:704–714. 23847141 10.1136/jmedgenet-2013-101604 PMC3786640

[3] Hertweck K.L. , Dasgupta S. The landscape of mtDNA modifications in cancer: a tale of two cities. Front. Oncol. 2017; 7:262. 29164061 10.3389/fonc.2017.00262 PMC5673620

[4] Ju Y.S. , Alexandrov L.B., Gerstung M., Martincorena I., Nik-Zainal S., Ramakrishna M., Davies H.R., Papaemmanuil E., Gundem G., Shlien A. et al. Origins and functional consequences of somatic mitochondrial DNA mutations in human cancer. Elife. 2014; 3:e02935. 10.7554/eLife.02935 PMC4371858 25271376

[5] Tuppen H.A.L. , Blakely E.L., Turnbull D.M., Taylor R.W. Mitochondrial DNA mutations and human disease. BBA - Bioenerg. 2009; 1797:113–128. 10.1016/j.bbabio.2009.09.005 19761752

[6] Brown W.M. , George M., Wilson A.C. Rapid evolution of animal mitochondrial DNA. Proc. Natl. Acad. Sci. U.S.A. 1979; 76:1967–1971. 109836 10.1073/pnas.76.4.1967 PMC383514

[7] Brown W.M. , Prager E.M., Wang A., Wilson A.C. Mitochondrial DNA sequences of primates: tempo and mode of evolution. J. Mol. Evol. 1982; 18:225–239. 6284948 10.1007/BF01734101

[8] Denver D.R. , Morris K., Lynch M., Vassilieva L.L., Thomas W.K. High direct estimate of the mutation rate in the mitochondrial genome of *Caenorhabditis elegans*. Science. 2000; 289:2342–2345. 11009418 10.1126/science.289.5488.2342

[9] Itsara L.S. , Kennedy S.R., Fox E.J., Yu S., Hewitt J.J., Sanchez-Contreras M., Cardozo-Pelaez F., Pallanck L.J. Oxidative stress is not a major contributor to somatic mitochondrial DNA mutations. PLoS Genet. 2014; 10:e1003974. 24516391 10.1371/journal.pgen.1003974 PMC3916223

[10] Kennedy S.R. , Salk J.J., Schmitt M.W., Loeb L.A. Ultra-sensitive sequencing reveals an age-related increase in somatic mitochondrial mutations that are inconsistent with oxidative damage. PLoS Genet. 2013; 9:e1003794. 24086148 10.1371/journal.pgen.1003794 PMC3784509

[11] Sanchez-Contreras M. , Sweetwyne M.T., Kohrn B.F., Tsantilas K.A., Fredrickson J., Whitson J.A., Campbell M.D., Rabinovitch P.S., Marcinek D.J., Kennedy S.R. A mutational gradient drives somatic mutation accumulation in mitochondrial DNA and influences germline polymorphisms and genome composition. Nucleic Acids Res. 2021; 49:11103–11118. 34614167 10.1093/nar/gkab901 PMC8565317

[12] Krasich R. , Copeland W.C. DNA polymerases in the mitochondria: a critical review of the evidence. Front. Biosci. (Landmark Ed.). 2017; 22:692–709. 27814640 10.2741/4510 PMC5485829

[13] Cline S.D. Mitochondrial DNA damage and its consequences for mitochondrial gene expression. Biochim. Biophys. Acta - Gene Regul. Mech. 2012; 1819:979–991. 10.1016/j.bbagrm.2012.06.002 PMC3412069 22728831

[14] Gustafson M.A. , Sullivan E.D., Copeland W.C. Consequences of compromised mitochondrial genome integrity. DNA Repair (Amst). 2020; 93:102916. 33087282 10.1016/j.dnarep.2020.102916 PMC7587307

[15] Valente W.J. , Ericson N.G., Long A.S., White P.A., Marchetti F., Bielas J.H. Mitochondrial DNA exhibits resistance to induced point and deletion mutations. Nucleic Acids Res. 2016; 44:8513–8524. 27550180 10.1093/nar/gkw716 PMC5062989

[16] Meyer J.N. , Leung M.C.K., Rooney J.P., Sendoel A., Hengartner M.O., Kisby G.E., Bess A.S. Mitochondria as a target of environmental toxicants. Toxicol. Sci. 2013; 134:1–17. 23629515 10.1093/toxsci/kft102 PMC3693132

[17] Meyer J.N. , Hartman J.H., Mello D.F. Mitochondrial toxicity. Toxicol. Sci. 2018; 162:15–23. 29340618 10.1093/toxsci/kfy008 PMC5837373

[18] Roubicek D.A. , de Souza-Pinto N.C. Mitochondria and mitochondrial DNA as relevant targets for environmental contaminants. Toxicology. 2017; 391:100–108. 28655544 10.1016/j.tox.2017.06.012

[19] Nunnari J. , Suomalainen A. Mitochondria: in sickness and in health. Cell. 2012; 148:1145–1159. 22424226 10.1016/j.cell.2012.02.035 PMC5381524

[20] Meyer J.N. , Leuthner T.C., Luz A.L. Mitochondrial fusion, fission, and mitochondrial toxicity. Toxicology. 2017; 391:42–53. 28789970 10.1016/j.tox.2017.07.019 PMC5681418

[21] Luz A.L. , Godebo T.R., Smith L.L., Leuthner T.C., Maurer L.L., Meyer J.N. Deficiencies in mitochondrial dynamics sensitize caenorhabditis elegans to arsenite and other mitochondrial toxicants by reducing mitochondrial adaptability. Toxicology. 2017; 387:81–94. 28602540 10.1016/j.tox.2017.05.018 PMC5535741

[22] Luz A.L. , Rooney J.P., Kubik L.L., Gonzalez C.P., Song D.H., Meyer J.N. Mitochondrial morphology and fundamental parameters of the mitochondrial respiratory chain are altered in *Caenorhabditis elegans* strains deficient in mitochondrial dynamics and homeostasis processes. PLoS One. 2015; 10:e0130940. 26106885 10.1371/journal.pone.0130940 PMC4480853

[23] Bess A.S. , Crocker T.L., Ryde I.T., Meyer J.N. Mitochondrial dynamics and autophagy aid in removal of persistent mitochondrial DNA damage in *Caenorhabditis elegans*. Nucleic Acids Res. 2012; 40:7916–7931. 22718972 10.1093/nar/gks532 PMC3439916

[24] Bess A.S. , Leung M.C.K., Ryde I.T., Rooney J.P., Hinton D.E., Meyer J.N. Effects of mutations in mitochondrial dynamics-related genes on the mitochondrial response to ultraviolet c radiation in developing *Caenorhabditis elegans*. Worm. 2013; 2:e23763. 24058863 10.4161/worm.23763 PMC3670464

[25] Jansen R.P.S. , De Boer K. The bottleneck: mitochondrial imperatives in oogenesis and ovarian follicular fate. Mol. Cell. Endocrinol. 1998; 145:81–88. 9922103 10.1016/s0303-7207(98)00173-7

[26] Bergstrom C.T. , Pritchard J. Germline bottlenecks and the evolutionary maintenance of mitochondrial genomes. Genetics. 1998; 149:2135–2146. 9691064 10.1093/genetics/149.4.2135 PMC1460283

[27] Cree L.M. , Samuels D.C., de Sousa Lopes S.C., Rajasimha H.K., Wonnapinij P., Mann J.R., Dahl H.-H.M., Chinnery P.F. A reduction of mitochondrial DNA molecules during embryogenesis explains the rapid segregation of genotypes. Nat. Genet. 2008; 40:249–254. 18223651 10.1038/ng.2007.63

[28] Cao L. , Shitara H., Sugimoto M., Hayashi J.-I., Abe K., Yonekawa H. New evidence confirms that the mitochondrial bottleneck is generated without reduction of mitochondrial DNA content in early primordial germ cells of mice. PLoS Genet. 2009; 5:e1000756. 19997484 10.1371/journal.pgen.1000756 PMC2777314

[29] Wai T. , Teoli D., Shoubridge E.A. The mitochondrial DNA genetic bottleneck results from replication of a subpopulation of genomes. Nat. Genet. 2008; 40:1484–1488. 19029901 10.1038/ng.258

[30] Mishra P. , Chan D.C. Mitochondrial dynamics and inheritance during cell division, development and disease. Nat Rev Mol Cell Biol. 2014; 15:634–646. 25237825 10.1038/nrm3877 PMC4250044

[31] Valenci I. , Yonai L., Bar-Yaacov D., Mishmar D., Ben-Zvi A. Parkin modulates heteroplasmy of truncated mtDNA in *Caenorhabditis elegans*. Mitochondrion. 2015; 20:64–70. 25462019 10.1016/j.mito.2014.11.001

[32] Haroon S. , Li A., Weinert J.L., Fritsch C., Ericson N.G., Alexander-Floyd J., Braeckman B.P., Haynes C.M., Bielas J.H., Gidalevitz T. et al. Multiple molecular mechanisms rescue mtDNA disease in C. elegans. Cell Rep. 2018; 22:3115–3125. 29562168 10.1016/j.celrep.2018.02.099 PMC6106782

[33] Katju V. , Bergthorsson U. Old trade, new tricks: insights into the spontaneous mutation process from the partnering of classical mutation accumulation experiments with high-throughput genomic approaches. Genome Biol. Evol. 2019; 11:136–165. 30476040 10.1093/gbe/evy252 PMC6330053

[34] Konrad A. , Thompson O., Waterston R.H., Moerman D.G., Keightley P.D., Bergthorsson U., Katju V. Mitochondrial mutation rate, spectrum and heteroplasmy in *Caenorhabditis elegans* spontaneous mutation accumulation lines of differing population size. Mol. Biol. Evol. 2017; 34:1319–1334. 28087770 10.1093/molbev/msx051 PMC5850408

[35] Wernick R.I. , Estes S., Howe D.K., Denver D.R. Paths of heritable mitochondrial DNA mutation and heteroplasmy in reference and gas-1 strains of *Caenorhabditis elegans*. Front. Genet. 2016; 7:51. 27148352 10.3389/fgene.2016.00051 PMC4829587

[36] Waneka G. , Svendsen J.M., Havird J.C., Sloan D.B. Mitochondrial mutations in *Caenorhabditis elegans* show signatures of oxidative damage and an AT-bias. Genetics. 2021; 219:iyab116. 34849888 10.1093/genetics/iyab116 PMC8633081

[37] Schmitt M.W. , Kennedy S.R., Salk J.J., Fox E.J., Hiatt J.B., Loeb L.A. Detection of ultra-rare mutations by next-generation sequencing. Proc. Natl. Acad. Sci. U.S.A. 2012; 109:14508–14513. 22853953 10.1073/pnas.1208715109 PMC3437896

[38] Valentine C.C. , Young R.R., Fielden M.R., Kulkarni R., Williams L.N., Li T., Minocherhomji S., Salk J.J. Direct quantification of in vivo mutagenesis and carcinogenesis using duplex sequencing. Proc. Natl. Acad. Sci. U.S.A. 2020; 117:33414–33425. 33318186 10.1073/pnas.2013724117 PMC7776782

[39] Abascal F. , Harvey L.M.R., Mitchell E., Lawson A.R.J., Lensing S.V., Ellis P., Russell A.J.C., Alcantara R.E., Baez-Ortega A., Wang Y. et al. Somatic mutation landscapes at single-molecule resolution. Nature. 2021; 593:405–410. 33911282 10.1038/s41586-021-03477-4

[40] Jin Y.H. , Clark A.B., Slebos R.J.C., Al-Refai H., Taylor J.A., Kunkel T.A., Resnick M.A., Gordenin D.A. Cadmium is a mutagen that acts by inhibiting mismatch repair. Nat. Genet. 2003; 34:326–329. 12796780 10.1038/ng1172 PMC2662193

[41] Liu J. , Qu W., Kadiiska M.B. Role of oxidative stress in cadmium toxicity and carcinogenesis. Toxicol. Appl. Pharmacol. 2009; 238:209–214. 19236887 10.1016/j.taap.2009.01.029 PMC4287357

[42] Chawanthayatham S. , Valentine C.C., Fedeles B.I., Fox E.J., Loeb L.A., Levine S.S., Slocum S.L., Wogan G.N., Croy R.G., Essigmann J.M. Mutational spectra of aflatoxin B1 in vivo establish biomarkers of exposure for human hepatocellular carcinoma. Proc. Natl. Acad. Sci. U.S.A. 2017; 114:E3101–E3109. 28351974 10.1073/pnas.1700759114 PMC5393230

[43] González-Hunt C.P. , Leung M.C.K., Bodhicharla R.K., McKeever M.G., Arrant A.E., Margillo K.M., Ryde I.T., Cyr D.D., Kosmaczewski S.G., Hammarlund M. et al. Exposure to mitochondrial genotoxins and dopaminergic neurodegeneration in *Caenorhabditis elegans*. PLoS One. 2014; 9:e114459. 25486066 10.1371/journal.pone.0114459 PMC4259338

[44] Leung M.C.K. , Goldstone J.V, Boyd W.A., Freedman J.H., Meyer J.N. *Caenorhabditis elegans* generates biologically relevant levels of genotoxic metabolites from aflatoxin B1 but not benzo(a)pyrene in vivo. Toxicol. Sci. 2010; 118:444–453. 20864627 10.1093/toxsci/kfq295 PMC2984530

[45] Niranjan A.B.G. , Bhat N.K., Avadhani N.G. Preferential attack of mitochondrial DNA by aflatoxin B1 during hepatocarcinogenesis. Science. 1982; 215:73–75. 6797067 10.1126/science.6797067

[46] Palikaras K. , Lionaki E., Tavernarakis N. Coordination of mitophagy and mitochondrial biogenesis during ageing in *C. elegans*. Nature. 2015; 521:525–528. 25896323 10.1038/nature14300

[47] Brenner S. The genetics of *Caenorhabditis elegans*. Genetics. 1974; 77:71–94. 4366476 10.1093/genetics/77.1.71 PMC1213120

[48] Williams P.L. , Dusenbery D.B. Using the nematode *Caenorhabditis elegans* to predict mammalian acute lethality to metallic salts. Toxicol. Ind. Health. 1988; 4:469–478. 3188044 10.1177/074823378800400406

[49] Boyd W.A. , McBride S.J., Rice J.R., Snyder D.W., Freedman J.H. A high-throughput method for assessing chemical toxicity using a *Caenorhabditis elegans* reproduction assay. Toxicol. Appl. Pharmacol. 2010; 245:153–159. 20206647 10.1016/j.taap.2010.02.014 PMC2871981

[50] Cothren S. , Meyer J., Hartman J. Blinded visual scoring of images using the Freely-available software blinder. Bio-Protocol. 2018; 8:e3103. 30761327 10.21769/BioProtoc.3103 PMC6370323

[51] Hartman J.H. , Smith L.L., Gordon K.L., Laranjeiro R., Driscoll M., Sherwood D.R., Meyer J.N. Swimming exercise and transient food deprivation in *Caenorhabditis elegans* promote mitochondrial maintenance and protect against chemical-induced mitotoxicity. Sci. Rep. 2018; 8:8359. 29844465 10.1038/s41598-018-26552-9 PMC5974391

[52] Luz A.L. , Smith L.L., Rooney J.P., Meyer J.N. Seahorse xfe 24 extracellular flux analyzer-based analysis of cellular respiration in *Caenorhabditis elegans*. Current Protocols in Toxicology. 2015; 66:Hoboken, NJ, USA John Wiley & Sons, Inc 25.7.1–25.7.15. 10.1002/0471140856.tx2507s66 PMC4632645 26523474

[53] Moore B.T. , Jordan J.M., Baugh L.R. WormSizer: High-throughput analysis of nematode size and shape. PLoS One. 2013; 8:e57142. 23451165 10.1371/journal.pone.0057142 PMC3579787

[54] Hodgkin J. , Barnes T.A. More is not better: brood size and population growth in a self-fertilizing nematode. Proc. R. Soc. London. Ser. B Biol. Sci. 1991; 246:19–24. 10.1098/rspb.1991.0119 1684664

[55] Gonzalez-Hunt C.P. , Rooney J.P., Ryde I.T., Anbalagan C., Joglekar R., Meyer J.N. PCR-Based analysis of mitochondrial DNA copy number, mitochondrial DNA damage, and nuclear DNA damage. Curr. Protoc. Toxicol. 2016; 67:20.11.1–20.11.25. 26828332 10.1002/0471140856.tx2011s67 PMC4928199

[56] Leuthner T.C. , Hartman J.H., Ryde I.T., Meyer J.N. PCR-Based determination of mitochondrial DNA copy number in multiple species. Methods Mol. Biol. 2021; 2310:91–111. 34096001 10.1007/978-1-0716-1433-4_8

[57] Denver D.R. , Feinberg S., Estes S., Thomas W.K., Lynch M. Mutation rates, spectra and hotspots in mismatch repair-deficient *Caenorhabditis elegans*. Genetics. 2005; 170:107–113. 15716493 10.1534/genetics.104.038521 PMC1449714

[58] Kennedy S.R. , Schmitt M.W., Fox E.J., Kohrn B.F., Salk J.J., Ahn E.H., Prindle M.J., Kuong K.J., Shen J.C., Risques R.A. et al. Detecting ultralow-frequency mutations by duplex sequencing. Nat. Protoc. 2014; 9:2586–2606. 25299156 10.1038/nprot.2014.170 PMC4271547

[59] Hoekstra J.G. , Hipp M.J., Montine T.J., Kennedy S.R. Mitochondrial DNA mutations increase in early stage alzheimer disease and are inconsistent with oxidative damage. Ann. Neurol. 2016; 80:301–306. 27315116 10.1002/ana.24709 PMC4982791

[60] Blokzijl F. , Janssen R., van Boxtel R., Cuppen E. MutationalPatterns: comprehensive genome-wide analysis of mutational processes. Genome Med. 2018; 10:33. 29695279 10.1186/s13073-018-0539-0 PMC5922316

[61] Martincorena I. , Raine K.M., Gerstung M., Dawson K.J., Haase K., Van Loo P., Davies H., Stratton M.R., Campbell P.J. Universal patterns of selection in cancer and somatic tissues. Cell. 2017; 171:1029–1041. 29056346 10.1016/j.cell.2017.09.042 PMC5720395

[62] Meier B. , Cooke S.L., Al. E *C. elegans* whole genome sequencing reveals mutational signatures related to carcinogens and DNA repair deficiency. Genome Res. 2014; 1:1624–1636. 10.1101/gr.175547.114 PMC4199376 25030888

[63] Denver D.R. , Dolan P.C., Wilhelm L.J., Sung W., Lucas-Lledó J.I., Howe D.K., Lewis S.C., Okamoto K., Thomas W.K., Lynch M. et al. A genome-wide view of *Caenorhabditis elegans* base-substitution mutation processes. Proc. Natl. Acad. Sci. U.S.A. 2009; 106:16310–16314. 19805298 10.1073/pnas.0904895106 PMC2752564

[64] Chen G. , Kroemer G., Kepp O. Mitophagy: an emerging role in aging and age-associated diseases. Front. Cell Dev. Biol. 2020; 8:200. 32274386 10.3389/fcell.2020.00200 PMC7113588

[65] Hanna R.A. , Quinsay M.N., Orogo A.M., Giang K., Rikka S., Gustafsson Å.B. Microtubule-associated protein 1 light chain 3 (LC3) interacts with bnip3 protein to selectively remove endoplasmic reticulum and mitochondria via autophagy. J. Biol. Chem. 2012; 287:19094–19104. 22505714 10.1074/jbc.M111.322933 PMC3365942

[66] Springer M.Z. , Macleod K.F. In brief: mitophagy: mechanisms and role in human disease. J. Pathol. 2016; 240:253–255. 27453450 10.1002/path.4774 PMC5071152

[67] Vassilieva L.L. , Hook A.M., Lynch M. The fitness effects of spontaneous mutations in *Caenorhabditis elegans*. Evolution. 2000; 54:1234–1246. 11005291 10.1111/j.0014-3820.2000.tb00557.x

[68] Estes S. , Phillips P.C., Denver D.R. Fitness recovery and compensatory evolution in natural mutant lines of *C. elegans*. Evolution. 2011; 65:2335–2344. 21790579 10.1111/j.1558-5646.2011.01276.x

[69] Ju Y.S. , Alexandrov L.B., Gerstung M., Martincorena I., Nik-Zainal S., Ramakrishna M., Davies H.R., Papaemmanuil E., Gundem G., Shlien A. et al. Origins and functional consequences of somatic mitochondrial DNA mutations in human cancer. Elife. 2014; 3:e02935. 10.7554/eLife.02935 PMC4371858 25271376

[70] Alexandrov L.B. , Kim J., Haradhvala N.J., Huang M.N., Tian Ng A.W., Wu Y., Boot A., Covington K.R., Gordenin D.A., Bergstrom E.N. et al. The repertoire of mutational signatures in human cancer. Nature. 2020; 578:94–101. 32025018 10.1038/s41586-020-1943-3 PMC7054213

[71] Keith N. , Jackson C.E., Glaholt S.P., Young K., Lynch M., Shaw J.R. Genome-Wide analysis of cadmium-induced, germline mutations in a long-term daphnia pulex mutation-accumulation experiment. Environ. Health Perspect. 2021; 129:107003. 34623885 10.1289/EHP8932 PMC8500294

[72] Volkova N.V. , Meier B., González-Huici V., Bertolini S., Gonzalez S., Vöhringer H., Abascal F., Martincorena I., Campbell P.J., Gartner A. et al. Mutational signatures are jointly shaped by DNA damage and repair. Nat. Commun. 2020; 11:2169. 32358516 10.1038/s41467-020-15912-7 PMC7195458

[73] Leuthner T.C. , Meyer J.N. Mitochondrial DNA mutagenesis: feature of and biomarker for environmental exposures and aging. Curr. Environ. Heal. Reports. 2021; 8:294–308. 10.1007/s40572-021-00329-1 PMC8826492 34761353

[74] Kauppila J.H.K. , Bonekamp N.A., Mourier A., Isokallio M.A., Just A., Kauppila T.E.S., Stewart J.B., Larsson N.G. Base-excision repair deficiency alone or combined with increased oxidative stress does not increase mtDNA point mutations in mice. Nucleic Acids Res. 2018; 46:6642–6649. 29860357 10.1093/nar/gky456 PMC6061787

[75] Meshnik L. , Bar-Yaacov D., Kasztan D., Neiger T., Cohen T., Kishner M., Valenci I., Dadon S., Klein C.J., Vance J.M. et al. Mutant *C. elegans* mitofusin leads to selective removal of mtDNA heteroplasmic deletions across generations to maintain fitness. BMC Biol. 2022; 20:40. 35139855 10.1186/s12915-022-01241-2 PMC8829988

[76] Pickrell A.M. , Huang C.H., Kennedy S.R., Ordureau A., Sideris D.P., Hoekstra J.G., Harper J.W., Youle R.J. Endogenous parkin preserves dopaminergic substantia nigral neurons following mitochondrial DNA mutagenic stress. Neuron. 2015; 87:371–381. 26182419 10.1016/j.neuron.2015.06.034 PMC4803114

[77] Haroon S. , Li A., Weinert J.L., Fritsch C., Ericson N.G., Alexander-Floyd J., Braeckman B.P., Haynes C.M., Bielas J.H., Gidalevitz T. et al. Multiple molecular mechanisms rescue mtDNA disease in *C. elegans*. Cell Rep. 2018; 22:3115–3125. 29562168 10.1016/j.celrep.2018.02.099 PMC6106782

[78] Schaack S. , Ho E.K.H., Macrae F. Disentangling the intertwined roles of mutation, selection and drift in the mitochondrial genome. Philos. Trans. R. Soc. B Biol. Sci. 2020; 375:20190173. 10.1098/rstb.2019.0173 PMC6939366 31787045

[79] Stewart J.B. , Freyer C., Elson J.L., Larsson N.-G. Purifying selection of mtDNA and its implications for understanding evolution and mitochondrial disease. Nat. Rev. Genet. 2008; 9:657–662. 18695671 10.1038/nrg2396

[80] Salk J.J. , Kennedy S.R. Next-Generation genotoxicology: using modern sequencing technologies to assess somatic mutagenesis and cancer risk. Environ. Mol. Mutagen. 2020; 61:135–151. 31595553 10.1002/em.22342 PMC7003768

[81] Schaack S. , Ho E.K.H., Macrae F. Disentangling the intertwined roles of mutation, selection and drift in the mitochondrial genome. Philos. Trans. R. Soc. B Biol. Sci. 2020; 375:20190173. 10.1098/rstb.2019.0173 PMC6939366 31787045

[82] Samstag C.L. , Hoekstra J.G., Huang C.H., Chaisson M.J., Youle R.J., Kennedy S.R., Pallanck L.J. Deleterious mitochondrial DNA point mutations are overrepresented in drosophila expressing a proofreading-defective DNA polymerase γ. PLoS Genet. 2018; 14:e1007805. 30452458 10.1371/journal.pgen.1007805 PMC6289449

[83] Jansson K. , Blomberg A., Sunnerhagen P., Alm Rosenblad M. Evolutionary loss of 8-oxo-G repair components among eukaryotes. Genome Integr. 2010; 1:12. 20809962 10.1186/2041-9414-1-12 PMC2941479

[84] Arczewska K.D. , Baumeier C., Kassahun H., SenGupta T., Bjørås M., Kuśmierek J.T., Nilsen H. *Caenorhabditis elegans* NDX-4 is a mutt-type enzyme that contributes to genomic stability. DNA Repair (Amst). 2011; 10:176–187. 21111690 10.1016/j.dnarep.2010.10.009

[85] Sanada U. , Yonekura S.I., Kikuchi M., Hashiguchi K., Nakamura N., Yonei S., Zhang-Akiyama Q.M. NDX-1 protein hydrolyzes 8-oxo-7, 8-dihydrodeoxyguanosine-5′- diphosphate to sanitize oxidized nucleotides and prevent oxidative stress in caenorhabditis elegans. J. Biochem. 2011; 150:649–657. 21873335 10.1093/jb/mvr107

[86] Christy S.F. , Wernick R.I., Lue M.J., Velasco G., Howe D.K., Denver D.R., Estes S. Adaptive evolution under extreme genetic drift in oxidatively stressed *Caenorhabditis elegans*. Genome Biol. Evol. 2017; 9:3008–3022. 29069345 10.1093/gbe/evx222 PMC5714194

[87] Degtyareva N.P. , Saini N., Sterling J.F., Placentra V.C., Klimczak L.J., Gordenin D.A., Doetsch P.W. Mutational signatures of redox stress in yeast single-strand DNA and of aging in human mitochondrial DNA share a common feature. PLoS Biol. 2019; 17:e3000263. 31067233 10.1371/journal.pbio.3000263 PMC6527239

[88] Arbeithuber B. , Hester J., Cremona M.A., Stoler N., Zaidi A., Higgins B., Anthony K., Chiaromonte F., Diaz J., Id K.D.M. Age-related accumulation of de novo mitochondrial mutations in mammalian oocytes and somatic tissues. PLoS Biol. 2020; 18:e3000745. 32667908 10.1371/journal.pbio.3000745 PMC7363077

[89] Kennedy S.R. , Salk J.J., Schmitt M.W., Loeb L.A. Ultra-Sensitive sequencing reveals an age-related increase in somatic mitochondrial mutations that are inconsistent with oxidative damage. PLoS Genet. 2013; 9:e1003794. 24086148 10.1371/journal.pgen.1003794 PMC3784509

[90] Lewis S.C. , Joers P., Willcox S., Griffith J.D., Jacobs H.T., Hyman B.C. A rolling circle replication mechanism produces multimeric lariats of mitochondrial DNA in *Caenorhabditis elegans*. PLoS Genet. 2015; 11:e1004985. 25693201 10.1371/journal.pgen.1004985 PMC4334201

[91] Gitschlag B.L. , Kirby C.S., Samuels D.C., Gangula R.D., Mallal S.A., Patel M.R. Homeostatic responses regulate selfish mitochondrial genome dynamics in *C.elegans*. Cell Metab. 2016; 24:91–103. 27411011 10.1016/j.cmet.2016.06.008 PMC5287496

[92] Lin Y.-F. , Schulz A.M., Pellegrino M.W., Lu Y., Shaham S., Haynes C.M. Maintenance and propagation of a deleterious mitochondrial genome by the mitochondrial unfolded protein response. Nature. 2016; 533:416–419. 27135930 10.1038/nature17989 PMC4873342

[93] Lee W.K. , Thévenod F. Cell Organelles as Targets of Mammalian Cadmium Toxicity. 2020; Berlin, Heidelberg Springer. 10.1007/s00204-020-02692-8 32206829

[94] World Health Organization Preventing disease through healthy environments. Exposure to Cadmium: A Major Public Health Concern. 2010; World Health Organization.

[95] Agency for Toxic Substances and Disease Registry Toxicological Profile for Cadmium. 2019; Atlanta, GA. 24049863

[96] Hsu T. , Tsai H.-T., Huang K.-M., Luan M.-C., Hsieh C.-R. Sublethal levels of cadmium down-regulate the gene expression of DNA mismatch recognition protein MutS homolog 6 (MSH6) in zebrafish (*Danio rerio*) embryos. Chemosphere. 2010; 81:748–754. 20696460 10.1016/j.chemosphere.2010.07.014

[97] Abbà S. , Vallino M., Daghino S., Di Vietro L., Borriello R., Perotto S. A PLAC8-containing protein from an endomycorrhizal fungus confers cadmium resistance to yeast cells by interacting with mlh3p. Nucleic Acids Res. 2011; 39:7548–7563. 21672957 10.1093/nar/gkr336 PMC3177179

[98] Sherrer S.M. , Penland E., Modrich P. The mutagen and carcinogen cadmium is a high-affinity inhibitor of the zinc-dependent mutlα endonuclease. Proc. Natl. Acad. Sci. U.S.A. 2018; 115:7314–7319. 29941579 10.1073/pnas.1807319115 PMC6048502

[99] Antoniali G. , Marcuzzi F., Casarano E., Tell G. Cadmium treatment suppresses DNA polymerase δ catalytic subunit gene expression by acting on the p53 and Sp1 regulatory axis. DNA Repair (Amst). 2015; 35:90–105. 26519823 10.1016/j.dnarep.2015.08.007

[100] Neeley W.L. , Delaney J.C., Henderson P.T., Essigmann J.M. In vivo bypass efficiencies and mutational signatures of the guanine oxidation products 2-aminoimidazolone and 5-guanidino-4-nitroimidazole. J. Biol. Chem. 2004; 279:43568–43573. 15299010 10.1074/jbc.M407117200

[101] Kino K. , Kawada T., Hirao-Suzuki M., Morikawa M., Miyazawa H. Products of oxidative guanine damage form base pairs with guanine. Int. J. Mol. Sci. 2020; 21:7645. 10.3390/ijms21207645 PMC7589758 33076559

[102] Kino K. , Sugasawa K., Mizuno T., Bando T., Sugiyama H., Akita M., Miyazawa H., Hanaoka F. Eukaryotic DNA polymerases α, β and ϵ incorporate guanine opposite 2,2,4-triamino-5(2H)-oxazolone. ChemBioChem. 2009; 10:2613–2616. 19780077 10.1002/cbic.200900492

[103] Hunter S.E. , Gustafson M.A., Margillo K.M., Lee S.A., Ryde I.T., Meyer J.N. In vivo repair of alkylating and oxidative DNA damage in the mitochondrial and nuclear genomes of wild-type and glycosylase-deficient *Caenorhabditis elegans*. DNA Repair (Amst). 2012; 11:857–863. 22959841 10.1016/j.dnarep.2012.08.002 PMC3484215

[104] SenGupta T. , Palikaras K., Esbensen Y.Q., Konstantinidis G., Galindo F.J.N., Achanta K., Kassahun H., Stavgiannoudaki I., Bohr V.A., Akbari M. et al. Base excision repair causes age-dependent accumulation of single-stranded DNA breaks that contribute to parkinson disease pathology. Cell Rep. 2021; 36:109668. 34496255 10.1016/j.celrep.2021.109668 PMC8441048

[105] Elsakrmy N. , Zhang-Akiyama Q.M., Ramotar D. The base excision repair pathway in the nematode *Caenorhabditis elegans*. Front. Cell Dev. Biol. 2020; 8:598860. 33344454 10.3389/fcell.2020.598860 PMC7744777

[106] Sanada Y. , Zhang-Akiyama Q.M. An increase of oxidised nucleotides activates DNA damage checkpoint pathway that regulates post-embryonic development in *Caenorhabditis elegans*. Mutagenesis. 2014; 29:107–114. 24435662 10.1093/mutage/get067 PMC3924892

[107] Vartanian V. , Minko I.G., Chawanthayatham S., Egner P.A., Lin Y.C., Earley L.F., Makar R., Eng J.R., Camp M.T., Li L. et al. NEIL1 protects against aflatoxin-induced hepatocellular carcinoma in mice. Proc. Natl. Acad. Sci. U.S.A. 2017; 114:4207–4212. 28373545 10.1073/pnas.1620932114 PMC5402411

[108] Lieber T. , Jeedigunta S.P., Palozzi J.M., Lehmann R., Hurd T.R. Mitochondrial fragmentation drives selective removal of deleterious mtDNA in the germline. Nature. 2019; 570:380–384. 31092924 10.1038/s41586-019-1213-4 PMC6614061

[109] Wang Q. , Jia F., Guo C., Wang Y., Zhang X., Cui Y., Song M., Cao Z., Li Y. PINK1/Parkin-mediated mitophagy as a protective mechanism against AFB1-induced liver injury in mice. Food Chem. Toxicol. 2022; 164:113043. 35447291 10.1016/j.fct.2022.113043

[110] Wang Y. , Song M., Wang Q., Guo C., Zhang J., Zhang X., Cui Y., Cao Z., Li Y. PINK1/Parkin-mediated mitophagy is activated to protect against AFB1-induced kidney damage in mice. Chem. Biol. Interact. 2022; 358:109884. 35304092 10.1016/j.cbi.2022.109884

[111] Pan Y.X. , Luo Z., Zhuo M.Q., Wei C.C., Chen G.H., Song Y.F. Oxidative stress and mitochondrial dysfunction mediated Cd-induced hepatic lipid accumulation in zebrafish danio rerio. Aquat. Toxicol. 2018; 199:12–20. 29604498 10.1016/j.aquatox.2018.03.017

[112] Zhang L. , Yang F., Li Y., Cao H., Huang A., Zhuang Y., Zhang C., Hu G., Mao Y., Luo J. et al. The protection of selenium against cadmium-induced mitophagy via modulating nuclear xenobiotic receptors response and oxidative stress in the liver of rabbits. Environ. Pollut. 2021; 285:117301. 34049137 10.1016/j.envpol.2021.117301

[113] Leung M.C.K. , Rooney J.P., Ryde I.T., Bernal A.J., Bess A.S., Crocker T.L., Ji A.Q., Meyer J.N. Effects of early life exposure to ultraviolet c radiation on mitochondrial DNA content, transcription, ATP production, and oxygen consumption in developing *Caenorhabditis elegans*. BMC Pharmacol. Toxicol. 2013; 14:9. 23374645 10.1186/2050-6511-14-9 PMC3621653

[114] Lujan S.A. , Longley M.J., Humble M.H., Lavender C.A., Burkholder A., Blakely E.L., Alston C.L., Gorman G.S., Turnbull D.M., McFarland R. et al. Ultrasensitive deletion detection links mitochondrial DNA replication, disease, and aging. Genome Biol. 2020; 21:248. 32943091 10.1186/s13059-020-02138-5 PMC7500033

[115] Chen Z. , Wang Z.H., Zhang G., Bleck C.K.E., Chung D.J., Madison G.P., Lindberg E., Combs C., Balaban R.S., Xu H. Mitochondrial DNA segregation and replication restrict the transmission of detrimental mutation. J. Cell Biol. 2020; 219:e201905160. 32375181 10.1083/jcb.201905160 PMC7337505

[116] Zhao L. Mitochondrial DNA degradation: a quality control measure for mitochondrial genome maintenance and stress response. Enzymes. 2019; 45:311–341. 31627882 10.1016/bs.enz.2019.08.004 PMC6932861

[117] Wang Y. , Zhang Y., Chen L., Liang Q., Yin X.-M., Miao L., Kang B.-H., Xue D. Kinetics and specificity of paternal mitochondrial elimination in *Caenorhabditis elegans*. Nat. Commun. 2016; 7:12569. 27581092 10.1038/ncomms12569 PMC5025750

[118] Lim Y. , Rubio-Peña K., Sobraske P.J., Molina P.A., Brookes P.S., Galy V., Nehrke K. Fndc-1 contributes to paternal mitochondria elimination in *C. elegans*. Dev Biol. 2019; 454:15–20. 31233739 10.1016/j.ydbio.2019.06.016 PMC6717525

[119] Wang S. , Tang M., Pei B., Xiao X., Wang J., Hang H., Wu L. Cadmium-Induced germline apoptosis in *Caenorhabditis elegans*: the roles of HUS1, p53, and MAPK signaling pathways. Toxicol. Sci. 2008; 102:345–351. 17728284 10.1093/toxsci/kfm220

[120] Feng W. , Xue K.S., Tang L., Williams P.L., Wang J. Aflatoxin B1-Induced developmental and DNA damage in *Caenorhabditis elegans*. Toxins (Basel). 2017; 9:9. 10.3390/toxins9010009 PMC5308242 28035971

[121] U.S. Environmental Protection Agency TSCA chemical substance inventory. 2021; https://www.epa.gov/tsca-inventory.

[122] Landrigan P.J. , Fuller R., Acosta N.J.R., Adeyi O., Arnold R., Basu N.(Nil), Baldé A.B., Bertollini R., Bose-O’Reilly S., Boufford J.I. et al. The lancet commission on pollution and health. Lancet. 2018; 391:462–512. 29056410 10.1016/S0140-6736(17)32345-0

[123] Langie S.A.S. , Koppen G., Desaulniers D., Al-Mulla F., Al-Temaimi R., Amedei A., Azqueta A., Bisson W.H., Brown D.G., Brunborg G. et al. Causes of genome instability: the effect of low dose chemical exposures in modern society. Carcinogenesis. 2015; 36(Suppl. 1):S61–S88. 26106144 10.1093/carcin/bgv031 PMC4565613

[124] Yousefzadeh M. , Henpita C., Vyas R., Soto-Palma C., Robbins P., Niedernhofer L. Dna damage—how and why we age?. Elife. 2021; 10:e62852. 33512317 10.7554/eLife.62852 PMC7846274

[125] Stewart J.B. , Chinnery P.F. Extreme heterogeneity of human mitochondrial DNA from organelles to populations. Nat. Rev. Genet. 2021; 22:106–118. 32989265 10.1038/s41576-020-00284-x

[126] Yadav N. , Chandra D. Mitochondrial DNA mutations and breast tumorigenesis. Biochim. Biophys. Acta. 2013; 1836:336–344. 24140413 10.1016/j.bbcan.2013.10.002 PMC3891589

[127] Kalsbeek A.M.F. , Chan E.K.F., Corcoran N.M., Hovens C.M., Hayes V.M. Mitochondrial genome variation and prostate cancer: a review of the mutational landscape and application to clinical management. Oncotarget. 2017; 8:71342–71357. 29050365 10.18632/oncotarget.19926 PMC5642640

[128] Kabekkodu S.P. , Bhat S., Mascarenhas R., Mallya S., Bhat M., Pandey D., Kushtagi P., Thangaraj K., Gopinath P.M., Satyamoorthy K. Mitochondrial DNA variation analysis in cervical cancer. Mitochondrion. 2014; 16:73–82. 23851045 10.1016/j.mito.2013.07.001

[129] Chan S.S.L. Inherited mitochondrial genomic instability and chemical exposures. Toxicology. 2017; 391:75–83. 28756246 10.1016/j.tox.2017.07.014 PMC5681375

[130] Zhao L. , Sumberaz P. Mitochondrial DNA damage: prevalence, biological consequence, and emerging pathways. Chem. Res. Toxicol. 2020; 33:2491–2502. 32486637 10.1021/acs.chemrestox.0c00083 PMC7572651

