# Supplementary material for: Resistance of mitochondrial DNA to cadmium and Aflatoxin B1 damage-induced germline mutation accumulation in C. elegans
Source: Nucleic Acids Res. 2022 Aug 10;50(15):8626–42. doi: 10.1093/nar/gkac666 (PMC9410910; doi:10.1093/nar/gkac666)
Supplement: gkac666_Supplemental_Files [file gkac666_supplemental_files.zip › Supplemental Files.pdf]

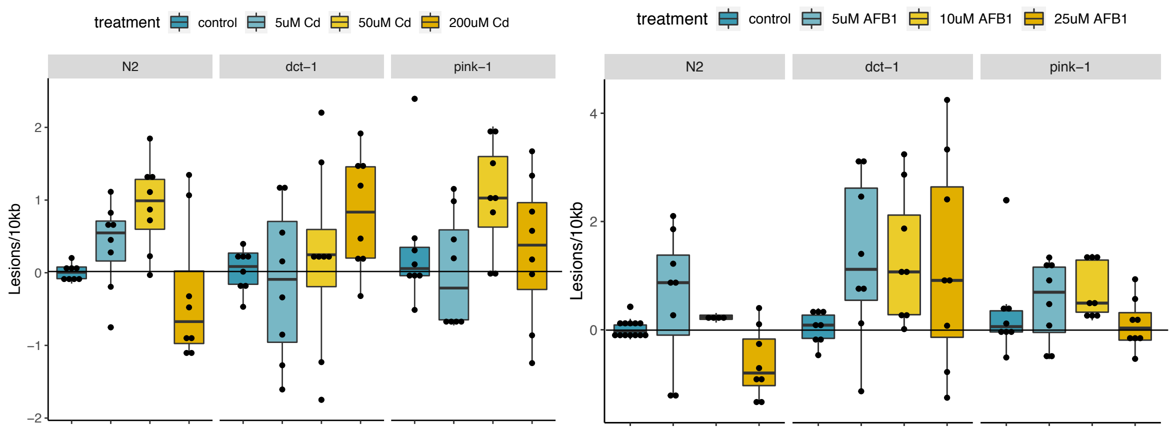

Figure S1. Wild-type (N2) and mitophagy mutant *C. elegans* accumulate mtDNA damage after exposure to $CdCl_2$ and AfB_1. We conducted a mtDNA damage dose-response for (A) $CdCl_2$ and (B) AfB_1 in wild-type, *dct-1*, and *pink-1* strains in order to determine a concentration for the MA line experiment that caused mtDNA lesions. Each dot is a biological replicate (N > 8 per concentration), with box-and-whisker plots to visualize distribution of mtDNA lesions (median and first and third quantiles). (A) There is a strain by treatment effect of Cd exposure ($P = 0.035$, two-way ANOVA). (B) There is a significant effect of treatment and strain in AfB_1 exposed *C. elegans*, though no significant interaction ($P = 0.19$, two-way ANOVA).

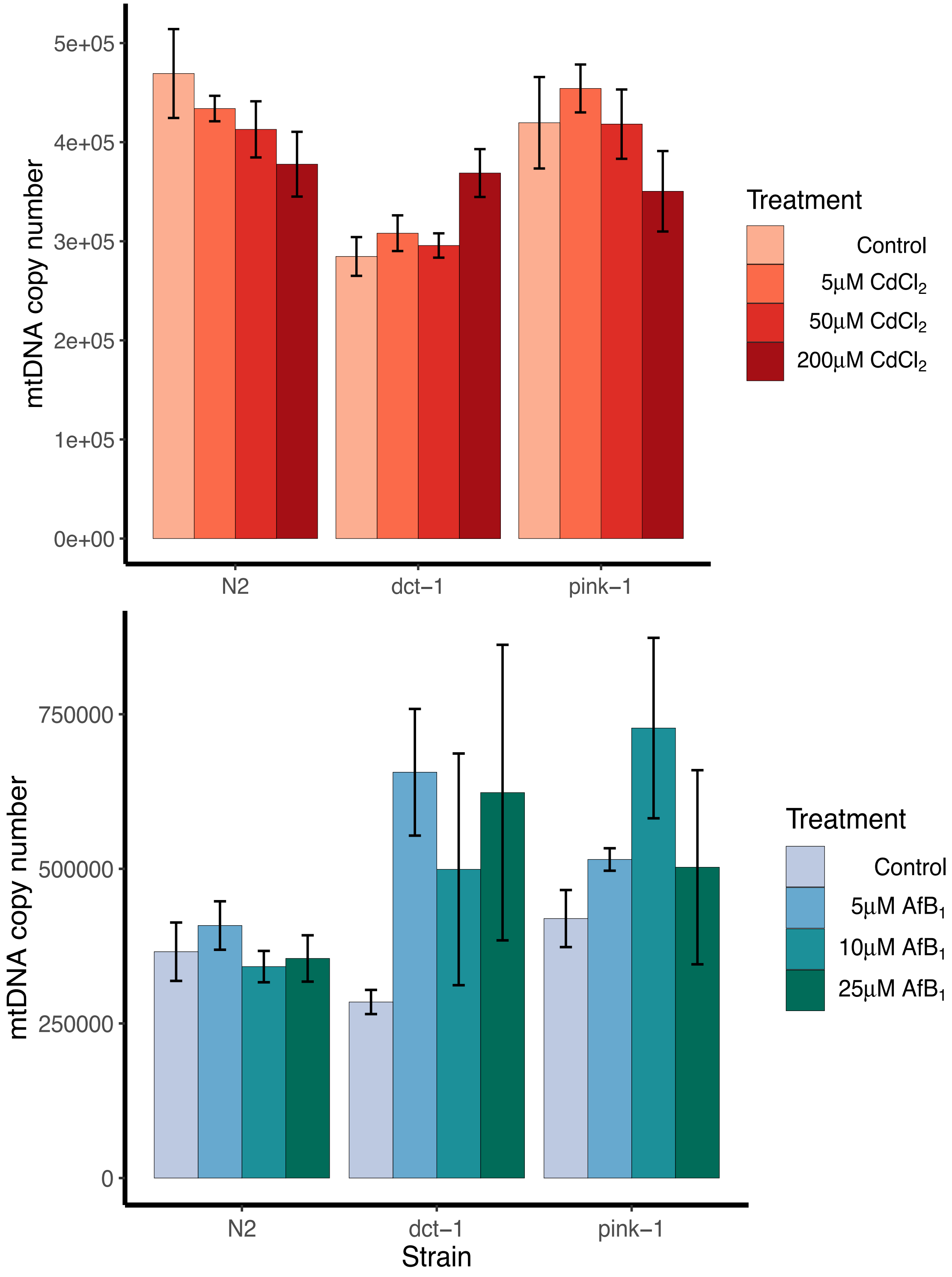

Figure S2. mtDNA copy number quantification in L4 C. elegans *in control, $CdCl_2$, and AfB_1 treatments.* Copy number was quantified using qPCR (see Methods) and is normalized and plotted as the mean mtDNA copy number per worm (line is standard error of the mean). The average of N2 control mtDNA

copy number is similar to previously published L4 mtDNA copy number values of ~$1.3x10^5$ (Lemire, 2005) and ~$5.6x10^5$ (Leung et al. 2013). There is a significant strain * treatment effect on mtDNA copy number after exposure to cadmium (*P* = 0.04, two-way ANOVA, N = 8 replicates per strain per treatment). However, the mtDNA copy number in the concentration that was used for the MA experiment and Duplex Sequencing (50μM) was not different from control levels in any strain. There is no significant strain * treatment effect on mtDNA copy number after exposure to aflatoxin (*P* = 0.4; two-way ANOVA; N = 4-13 replicates per strain per treatment).

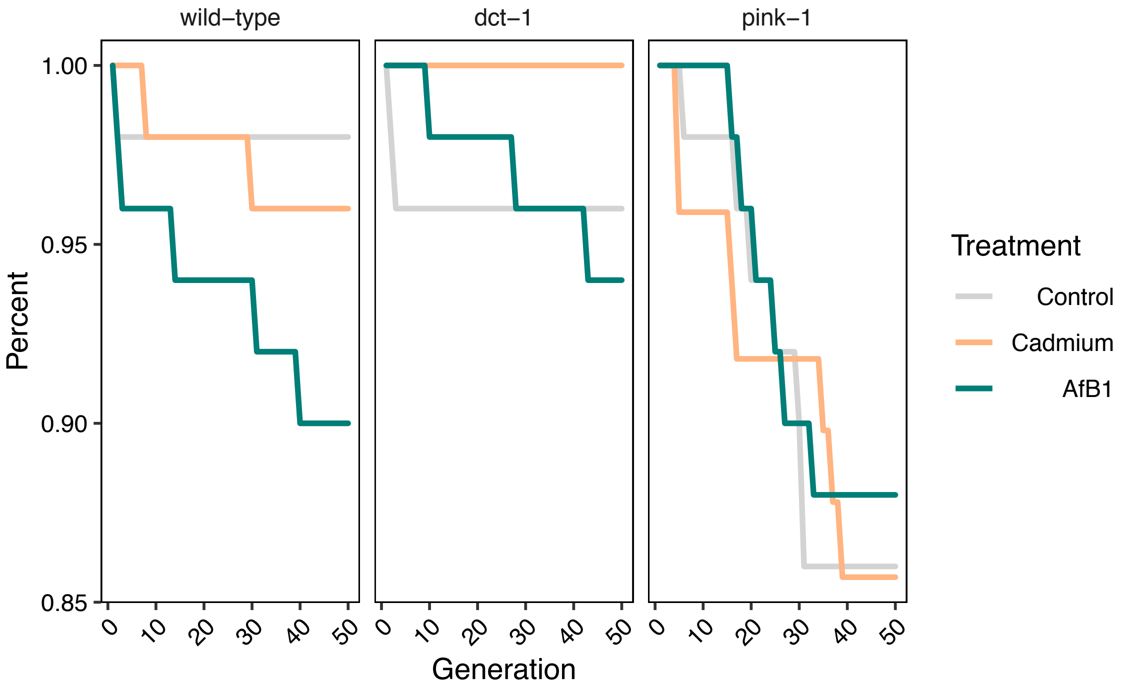

Figure S3. Mutation accumulation line extinction rates. The percentage of MA lines that were present at each generation out of 50 total MA lines per strain per treatment at G0. Y-axis begins at 85% survival. On average, 95.5% of MA lines were present at the end of the MA experiment (G50), though *pink-1* mutants had slightly higher extinction rates than wild-type and *dct-1* MA lines. A MA line was considered extinct if two back-up individual nematodes failed to produce offspring.

Table S1. Single nucleotide polymorphisms fixed in all 96 MA lines. These SNPS were not included in frequency calculations.

Position	Ref	Alt
8429	A	G
12392	C	T
12998	C	T

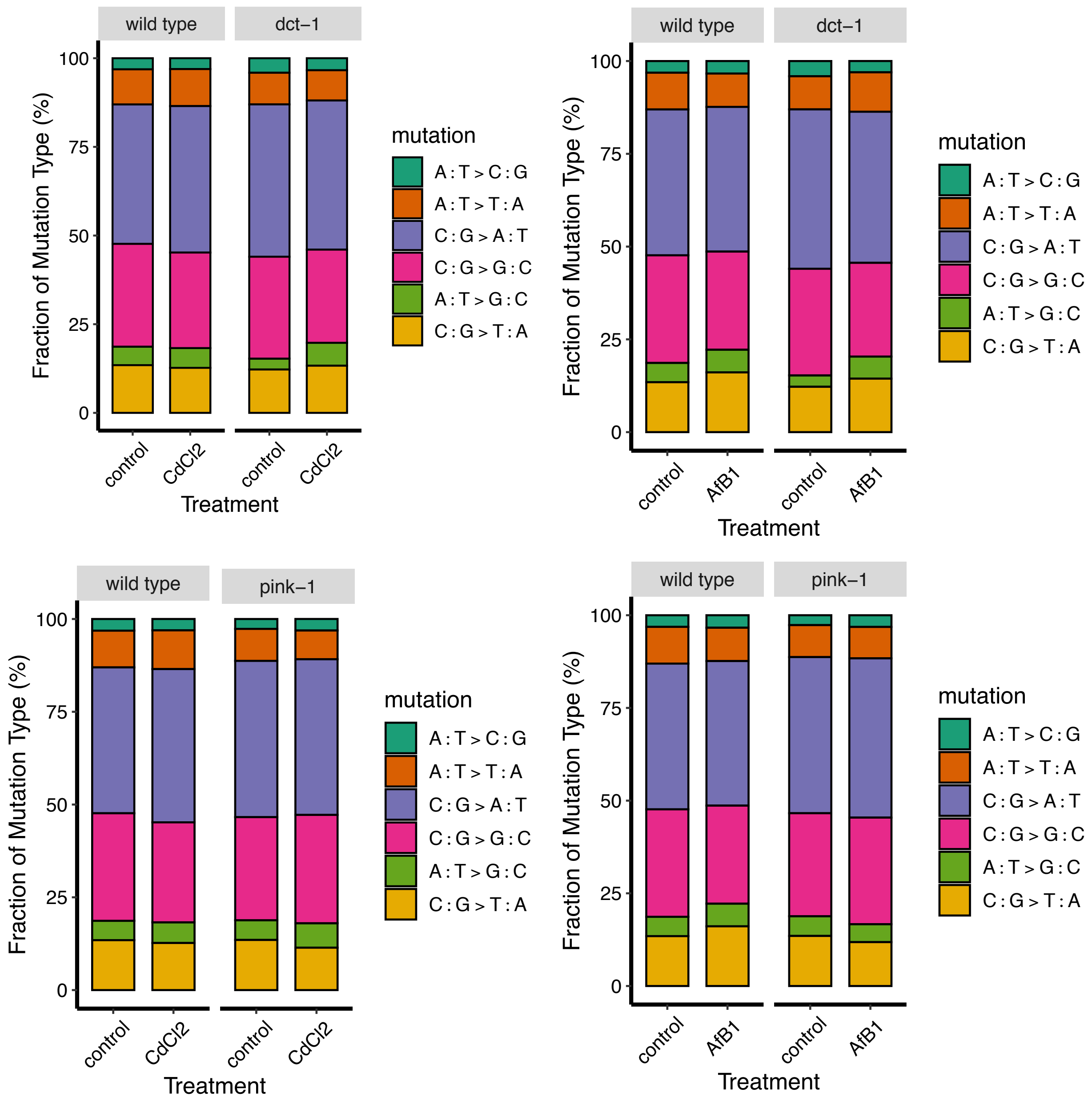

Figure S4. Proportion of each six possible single nucleotide substitution mutations. Composition (percentage) of each mutation type in wild-type and *dct-1* mutants in (A) cadmium and (B) aflatoxin conditions, and wild-type and *pink-1* mutants in (C) cadmium and (D) aflatoxin conditions. There is no statistical difference in the proportion of mutations between strains and no effect of treatment (Fisher's Exact Test). A majority of mutations are C:G → A:T and C:G → G:C transition mutations.

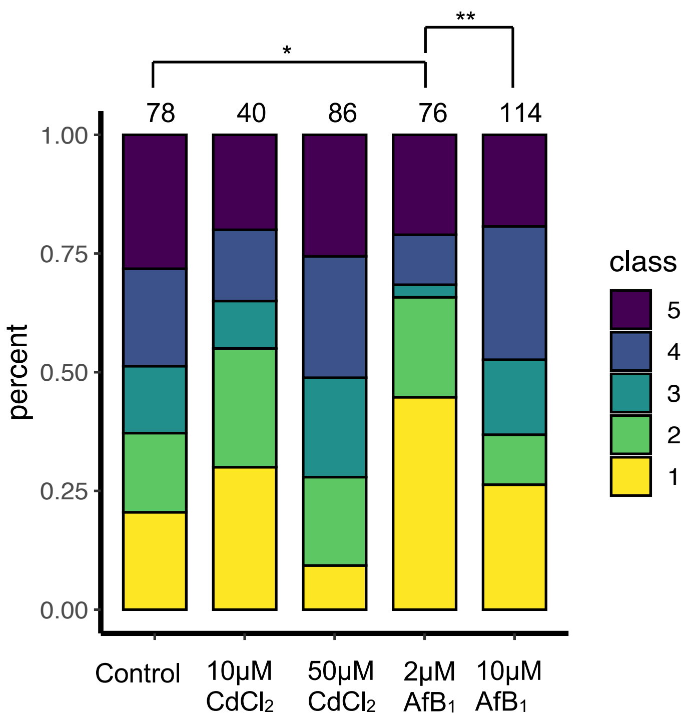

Figure S5. Effect of cadmium and aflatoxin exposure on C. elegans *body wall muscle mitochondrial morphology.* To assess effects of exposure on mitochondrial morphology, wild-type *C. elegans* harboring an extrachromosomal array Pmyo-3::GFP; which expresses GFP in the mitochondrial matrix of body wall muscle cells, were exposed to various concentrations of $CdCl_2$ and AfB_1. Images were taken on a Keyence, and analyzed in ImageJ using the Blinder Software (2). Blinded images were analyzed qualitatively by the established classification system as previously described (see Hartman et al. 2018 for representative class images and a detailed description) (3), with Class 1 indicating highly networked, fused mitochondrial morphology, and Class 5 indicating extremely fragmented mitochondrial morphology. Two experimental replicates were conducted, and the total number of individuals analyzed is displayed above each stacked bar plot. There were significantly fewer individuals with fragmented mitochondrial after exposure to 2μM AfB_1 compared to control (***P* = 0.002; Fisher's Exact Test, Bonferroni Multiple Comparisons Correction). This suggests that mitochondria are undergoing fusion in response to a low level of stress, and fission at the higher level of AfB_1 exposure (****P* < 0.0002; Fisher's Exact Test, Bonferroni Multiple Comparisons Correction). There is a trend towards a change in mitochondrial dynamics after exposure to low and high levels of $CdCl_2$ ($P = 0.02$; Fisher's Exact Test, Bonferroni Multiple Comparisons Correction), though not significant. Adjusted *alpha* = 0.005.

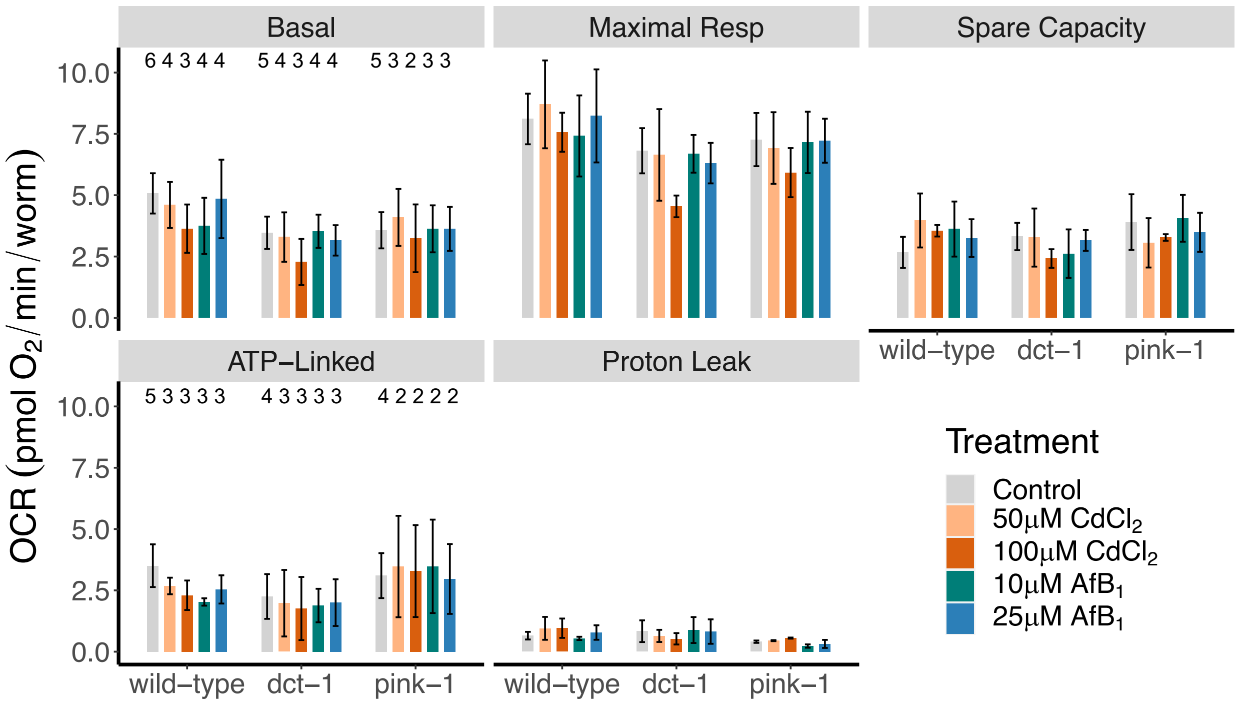

Figure S6. Effects of cadmium and aflatoxin exposure on mitochondrial function in wild-type, dct-1*, and* pink-1 C. elegans. Age-synchronized L1 *C. elegans* were transferred to plates seeded with OP50 containing 50μM and 100μM $CdCl_2$, or 10μM and 25μM AfB_1. Approximately 48 hours later, L4 *C. elegans* were washed off the plates and respiration parameters were quantified using the Seahorse Extracellular Flux Bioanalyzer, as previously described (4). The number of individual nematodes per well were counted, such that the OCR measurements were normalized per worm. The mean of technical replicates per plate was determined. Two to six independent experiments were conducted per strain per treatment. N (labeled on plot) is the same for Basal, Maximal Respiration, and Spare Capacity, and the same for ATP-Linked and Proton Leak. Two-way ANOVAs were run for each chemical in order to compare cadmium to control and aflatoxin to control across three strains. There was no significant strain * treatment interaction of cadmium compared to control in any respiration parameter (*P* = 0.97, 0.97, 0.71, 0.97, 0.83, Basal, Maximal Respiration, and Spare Capacity, ATP-Linked, Proton Leak, respectively; two-way ANOVA) or aflatoxin compared to control in any respiration parameter (*P* = 0.91, 0.99, 0.87, 0.90, 0.99, Basal, Maximal Respiration, and Spare Capacity, ATP-Linked, Proton Leak, respectively; two-way ANOVA).

Table S2. Overall mtDNA SNM frequencies. Duplex sequencing was conducted on 9-14 MA lines per strain per treatment. There was no significant Strain x Treatment interaction in the frequency of mtDNA SNMs after exposure to 50μM $CdCl_2$ or 10μM AfB_1 in wild-type compared to mitophagy mutants *dct-1* and *pink-1* (two-way ANOVA, P = 0.3).

Strain	Treatment	N	Mean overall SNM Frequency (10^{-7})
wild-type	Control	11	10.14
wild-type	50μM $CdCl_2$	14	9.75
wild-type	10μM AfB_1	10	9.47
dct-1	Control	9	8.93
dct-1	50μM $CdCl_2$	11	9.74
dct-1	10μM AfB_1	11	8.21
pink-1	Control	9	9.09
pink-1	50μM $CdCl_2$	11	9.16
pink-1	10μM AfB_1	10	11.63

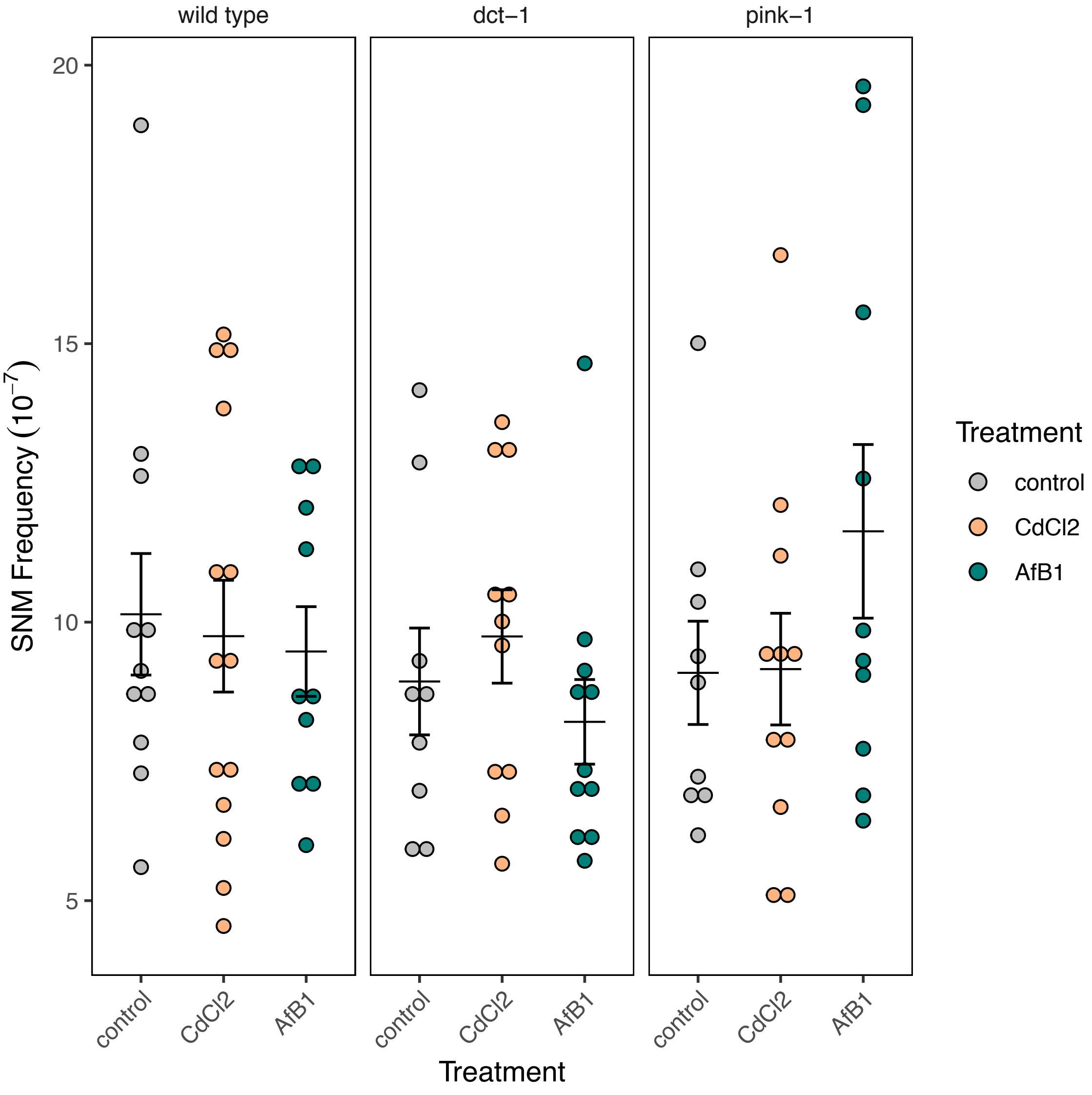

Figure S7. Overall mtDNA SNM frequencies. Overall SNM frequencies were determined by Duplex Sequencing after 50 generations of mutation accumulation in wild-type and two mitophagy-deficient mutants, *dct-1* and *pink-1*, *C. elegans* in control conditions (gray dots), 50μM $CdCl_2$ (gold dots), and 10μM AfB_1 (green dots). There was no significant effect of either 50μM $CdCl_2$ or 10μM AfB_1 on overall mtDNA mutation frequency compared to control between strains (two-way ANOVA, P = 0.3). Horizontal lines indicate mean values and error bars represent standard error of the mean.

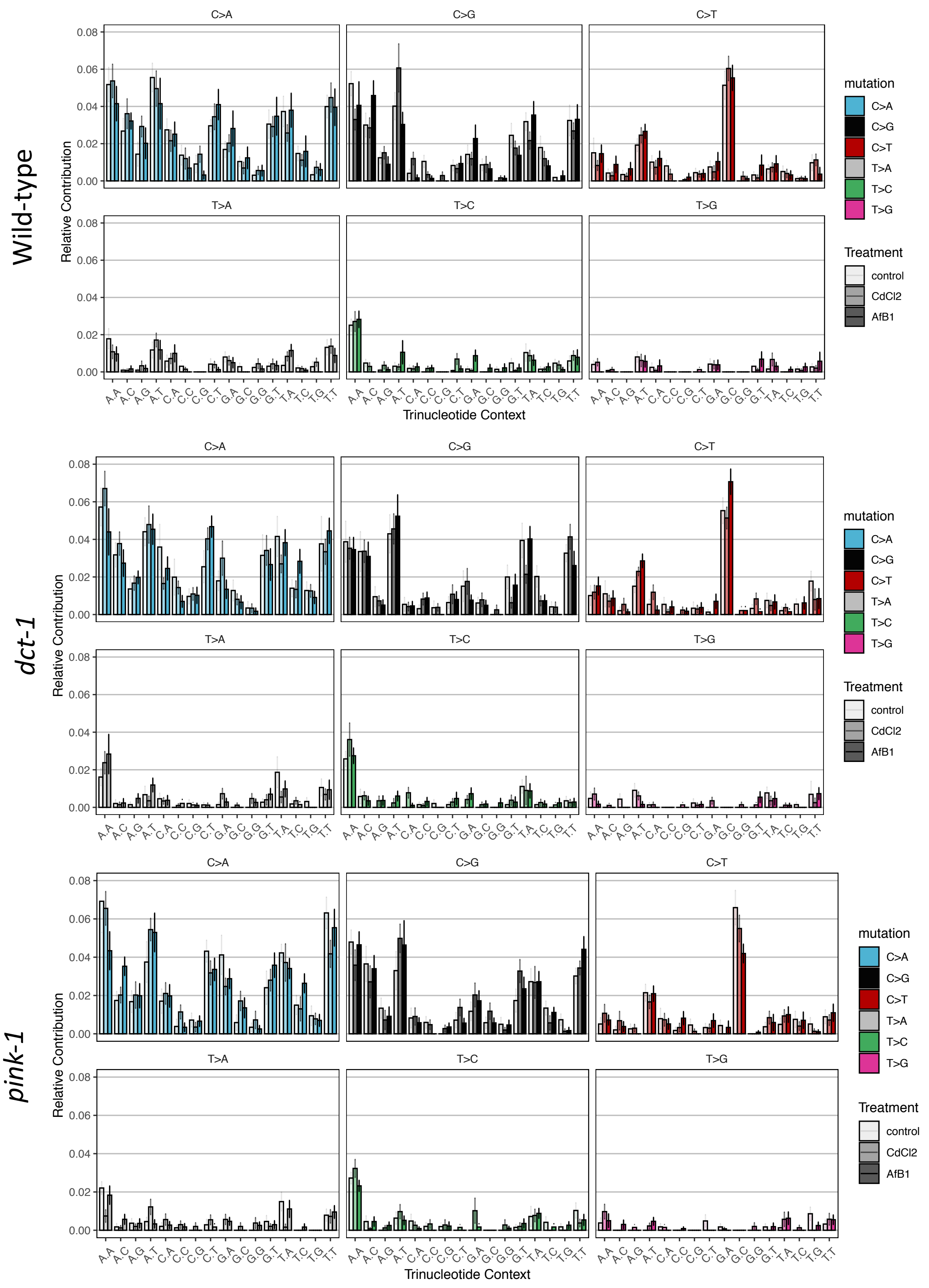
Wild-type
dct-1
pink-1
C>A
C>G
C>T
T>A
T>C
T>G
Relative Contribution
0.00
0.02
0.04
0.06
0.08
A.A A.C A.G A.T C.A C.C C.G C.T G.A G.C G.G G.T T.A T.C T.G T.T
Trinucleotide Context
mutation
C>A
C>G
C>T
T>A
T>C
T>G
Treatment
control
CdCl2
AfB1

Figure S8. C. elegans *mtDNA trinucleotide context mutational signatures in wild-type,* dct-1*, and* pink-1*s in control conditions and $CdCl_2$ and AfB_1 treatment.* Contributions of each of the 96 possible trinucleotide mutations were determined for each MA line. Bar graphs show the mean relative contribution and standard error of each trinucleotide context mutation, with each mutation represented by a different color and control, $CdCl_2$, and AfB_1 treatment as increasing color hues.

Table S3. Cosine similarity values of 96 single nucleotide mutational signature across MA lines. Total mutations within strain*treatment were combined into one VCF file and the R software *MutationalPatterns* was used to create a cosine similarity matrix between all nine MA lines. All signatures shared high similarity with cosine values greater than 0.9.

		Wild-type			*dct-1*			*pink-1*		
		Control	$CdCl_2$	AfB_1	Control	$CdCl_2$	AfB_1	Control	$CdCl_2$	AfB_1
Wild-type	Control	1.000	0.952	0.946	0.960	0.933	0.958	0.947	0.950	0.962
	$CdCl_2$	0.952	1.000	0.906	0.927	0.934	0.934	0.941	0.954	0.955
	AfB_1	0.946	0.906	1.000	0.953	0.940	0.946	0.938	0.938	0.947
dct-1	Control	0.960	0.927	0.953	1.000	0.931	0.964	0.936	0.935	0.957
	$CdCl_2$	0.933	0.934	0.940	0.931	1.000	0.936	0.926	0.950	0.949
	AfB_1	0.958	0.934	0.946	0.964	0.936	1.000	0.941	0.936	0.963
pink-1	Control	0.947	0.941	0.938	0.936	0.926	0.941	1.000	0.942	0.946
	$CdCl_2$	0.950	0.954	0.938	0.935	0.950	0.936	0.942	1.000	0.955
	AfB_1	0.962	0.955	0.947	0.957	0.949	0.963	0.946	0.955	1.000

Table S4. Cosine similarity values of MA lines to COSMIC Single Base Substitution Signatures. Using the R software package *Mutational Patterns* we investigated the similarity of MA lines to known mutational signatures. Cells in the table are colored by cosine similarity value, with those more similar to a SBS signature in red, and those less similar in blue. Overall, there is variation in similarity compared to known signatures, though these do not vary between strain and treatment.

	Wild-type			*dct-1*			*pink-1*			
SBS Signature	Control	$CdCl_2$	AfB_1	Control	$CdCl_2$	AfB_1	Control	$CdCl_2$	AfB_1	Mean
SBS1	0.032	0.025	0.066	0.033	0.039	0.035	0.043	0.038	0.018	0.034
SBS2	0.064	0.087	0.076	0.115	0.054	0.084	0.063	0.079	0.118	0.085
SBS3	0.661	0.651	0.678	0.639	0.648	0.655	0.610	0.658	0.655	0.644
SBS4	0.606	0.605	0.603	0.633	0.618	0.624	0.600	0.594	0.599	0.611
SBS5	0.446	0.450	0.516	0.434	0.463	0.470	0.451	0.465	0.465	0.458
SBS6	0.074	0.074	0.127	0.074	0.086	0.088	0.086	0.079	0.054	0.078
SBS7a	0.070	0.082	0.090	0.082	0.063	0.081	0.065	0.087	0.121	0.083
SBS7b	0.111	0.103	0.107	0.100	0.072	0.096	0.082	0.093	0.124	0.094
SBS7c	0.194	0.228	0.194	0.186	0.146	0.187	0.172	0.153	0.173	0.170
SBS7d	0.129	0.114	0.137	0.115	0.109	0.115	0.108	0.109	0.116	0.112
SBS8	0.648	0.661	0.653	0.657	0.648	0.650	0.627	0.644	0.642	0.645
SBS9	0.307	0.338	0.353	0.331	0.290	0.347	0.336	0.315	0.317	0.323
SBS10a	0.345	0.420	0.362	0.356	0.315	0.381	0.467	0.327	0.393	0.373
SBS10b	0.092	0.109	0.110	0.143	0.086	0.132	0.132	0.100	0.120	0.119
SBS11	0.099	0.100	0.138	0.120	0.105	0.132	0.089	0.100	0.117	0.111
SBS12	0.157	0.169	0.202	0.136	0.175	0.166	0.172	0.176	0.149	0.162
SBS13	0.385	0.326	0.445	0.431	0.424	0.427	0.345	0.377	0.428	0.405
SBS14	0.433	0.457	0.445	0.414	0.488	0.463	0.457	0.403	0.442	0.444
SBS15	0.109	0.109	0.146	0.126	0.116	0.127	0.117	0.115	0.094	0.116
SBS16	0.235	0.233	0.280	0.218	0.276	0.236	0.245	0.280	0.255	0.252
SBS17a	0.020	0.062	0.038	0.040	0.044	0.043	0.027	0.027	0.018	0.033
SBS17b	0.019	0.031	0.028	0.034	0.032	0.042	0.041	0.015	0.019	0.030
SBS18	0.610	0.618	0.655	0.650	0.631	0.644	0.719	0.626	0.632	0.651
SBS19	0.112	0.112	0.149	0.102	0.094	0.105	0.095	0.099	0.133	0.105
SBS20	0.253	0.262	0.277	0.234	0.305	0.277	0.243	0.226	0.212	0.250
SBS21	0.082	0.078	0.136	0.065	0.103	0.107	0.083	0.111	0.071	0.090
SBS22	0.137	0.156	0.142	0.149	0.120	0.153	0.121	0.120	0.126	0.132
SBS23	0.092	0.093	0.127	0.090	0.084	0.110	0.090	0.091	0.094	0.093
SBS24	0.439	0.427	0.457	0.465	0.472	0.462	0.477	0.459	0.466	0.467
SBS25	0.421	0.441	0.472	0.426	0.428	0.453	0.417	0.416	0.423	0.427
SBS26	0.117	0.129	0.165	0.098	0.151	0.132	0.133	0.146	0.113	0.129
SBS28	0.055	0.056	0.076	0.069	0.056	0.086	0.053	0.060	0.064	0.065
SBS29	0.569	0.563	0.600	0.611	0.635	0.590	0.625	0.603	0.594	0.610
SBS30	0.135	0.129	0.193	0.124	0.131	0.149	0.108	0.130	0.150	0.132
SBS31	0.223	0.217	0.227	0.197	0.191	0.215	0.201	0.205	0.232	0.207
SBS32	0.212	0.212	0.302	0.225	0.254	0.259	0.204	0.220	0.236	0.233
SBS33	0.104	0.106	0.097	0.075	0.079	0.091	0.086	0.079	0.102	0.085
SBS34	0.169	0.188	0.186	0.183	0.113	0.180	0.158	0.119	0.146	0.150
SBS35	0.402	0.404	0.411	0.402	0.412	0.407	0.364	0.402	0.415	0.400
SBS36	0.571	0.557	0.608	0.600	0.557	0.618	0.670	0.549	0.592	0.598

SBS37	0.280	0.277	0.310	0.254	0.283	0.274	0.292	0.286	0.265	0.276
SBS38	0.283	0.292	0.292	0.345	0.288	0.308	0.248	0.269	0.265	0.287
SBS39	0.679	0.640	0.666	0.643	0.641	0.652	0.619	0.671	0.671	0.650
SBS40	0.694	0.695	0.734	0.696	0.694	0.707	0.681	0.696	0.711	0.697
SBS41	0.317	0.344	0.366	0.344	0.282	0.337	0.314	0.297	0.324	0.316
SBS42	0.263	0.269	0.303	0.276	0.275	0.309	0.274	0.260	0.271	0.277
SBS44	0.252	0.256	0.314	0.218	0.275	0.272	0.244	0.229	0.209	0.241
SBS84	0.215	0.208	0.265	0.207	0.219	0.226	0.201	0.244	0.220	0.219
SBS85	0.306	0.313	0.305	0.289	0.259	0.320	0.293	0.244	0.273	0.280
SBS86	0.542	0.526	0.537	0.531	0.544	0.523	0.493	0.560	0.547	0.533
SBS87	0.156	0.156	0.174	0.164	0.166	0.152	0.176	0.163	0.152	0.162
SBS88	0.189	0.198	0.247	0.170	0.182	0.217	0.212	0.210	0.198	0.198
SBS89	0.624	0.618	0.668	0.617	0.637	0.640	0.591	0.629	0.623	0.623
SBS90	0.127	0.173	0.169	0.186	0.115	0.168	0.157	0.091	0.155	0.145

Table S5. Cosine similarity values to COSMIC Single Base Substitution Signatures. The mean cosine similarity value across all nine MA lines was determined for each of the 54 SBS Signatures from the COSMIC database (Table S4). Here the SBS signatures are ranked from high (similar) to low (not similar) cosine similarity values. There are 10 mutational signatures that have a cosine similarity value >0.5. The proposed etiology, if known, is listed.

SBS Signature	Mean Cosine Similarity Value	Proposed Etiology
SBS40	0.701	unknown (age)
SBS39	0.654	unknown
SBS3	0.651	Defective homologous recombination-based DNA damage repair, abnormal double strand break repair
SBS8	0.648	unknown
SBS18	0.643	**ROS**
SBS89	0.627	unknown
SBS4	0.609	Tobacco chewing
SBS29	0.599	Tobacco smoking
SBS36	0.591	Defective base excision repair, including DNA damage due to reactive oxygen species, due to biallelic germline or somatic *MUTYH* mutations.
SBS86	0.534	Unknown chemotherapy treatment
SBS5	0.462	Unknown (clock-like signature)
SBS24	0.458	**Aflatoxin exposure**
SBS14	0.445	Pol Epsilon mutation and defective MMR
SBS25	0.433	Chemotherapy
SBS35	0.402	Platinum chemotherapy
SBS13	0.399	APOBEC deaminases
SBS10a	0.374	Pol Epsilon exonuclease domain mutations
SBS9	0.326	Pol eta hypermutation
SBS41	0.325	unknown
SBS85	0.289	Indirect effects of activation-induced cytidine deamination
SBS38	0.288	UV (indirect)
SBS37	0.280	unknown
SBS42	0.278	Haloalkane exposure
SBS20	0.254	Defective MMR and POLD1 mutations
SBS44	0.252	Defective MMR
SBS16	0.251	unknown
SBS32	0.236	Azathioprine treatment
SBS84	0.223	Activation-induced cytidine deamination
SBS31	0.212	Platinum chemotherapy
SBS88	0.203	Colibactin exposure
SBS7c	0.182	UV exposure
SBS12	0.167	unknown
SBS87	0.162	Thiopurine chemotherapy
SBS34	0.160	unknown
SBS90	0.149	Duocarmycin exposure
SBS30	0.139	Defective BER (NTHL-1 mutations)
SBS22	0.136	Aristolochic Acid exposure
SBS26	0.132	Defective MMR
SBS15	0.118	Defective MMR

SBS7d	0.117	UV exposure
SBS10b	0.114	Pol Epsilon exonuclease domain mutations
SBS19	0.111	unknown
SBS11	0.111	Temozolomide treatment
SBS7b	0.099	UV exposure
SBS23	0.097	unknown
SBS21	0.093	Defective MMR
SBS33	0.091	unknown
SBS6	0.083	Defective MMR
SBS7a	0.082	UV exposure
SBS2	0.082	APOBEC deaminases
SBS28	0.064	unknown
SBS1	0.037	5-methylcytosine deamination
SBS17a	0.035	unknown
SBS17b	0.029	unknown

Table S6. Mutation accumulation lines are under neutral evolution. R package *dNdScv* was used to calculate the dN/dS ratio across wild-type, *dct-1*, and *pink-1* MA lines across control, cadmium, and aflatoxin treatment. Wmis = missense mutations, wnon = nonsense mutations, wall = all nonsynonymous mutations. All predicted global dN/dS values across all strains/treatments are close to 1, suggesting that the *C. elegans* mitochondrial genome is under neutral evolution in the MA experimental design.

Strain	Treatment	Name	Maximum likelihood model	CI (low)	CI (high)
wildtype	control	wmis	0.924	0.702	1.216
		wnon	0.908	0.542	1.520
		wall	0.924	0.702	1.216
wildtype	$CdCl_2$	wmis	1.159	0.887	1.515
		wnon	0.958	0.579	1.585
		wall	1.155	0.884	1.509
wildtype	AfB_1	wmis	1.1231009	0.8322949	1.515515
		wnon	0.7382623	0.3984926	1.367732
		wall	1.1151789	0.8263014	1.505049
dct-1	control	wmis	0.956759	0.6895992	1.32742
		wnon	0.6145301	0.3173671	1.189938
		wall	0.9465993	0.6821831	1.313504
dct-1	$CdCl_2$	wmis	0.9454341	0.7281853	1.227498
		wnon	0.7091876	0.3868122	1.300236
		wall	0.942165	0.7256459	1.223289
dct-1	AfB_1	wmis	1.0212917	0.7568162	1.37819
		wnon	0.8483719	0.4574501	1.573362
		wall	1.0180673	0.7544422	1.373811
pink-1	control	wmis	0.812797	0.600292	1.100529
		wnon	0.5416891	0.2885511	1.016898
		wall	0.8070964	0.5959419	1.093067
pink-1	$CdCl_2$	wmis	1.209978	0.8946023	1.636534
		wnon	1.209296	0.6848774	2.135269
		wall	1.209968	0.8946646	1.636394
pink-1	AfB_1	wmis	1.0284231	0.7750509	1.364625
		wnon	0.6751625	0.3787332	1.203603
		wall	1.0195962	0.7683399	1.353016

Table S7. Internal body burden of $CdCl_2$ in wild-type C. elegans. We measured the body burden of *$CdCl_2$* in adult *C. elegans* after a chronic exposure to 50µM $CdCl_2$. The actual concentration of Cd in the OP50 food source was slightly lower than 50µM (28 µM). However, the geometric mean of the internal concentration detected in three biological replicates averaged to 0.54 µg/L, which is comparable to human blood Cd levels (1), and 4-fold greater than control *C. elegans* samples.

			$[Cd]_{ICP}$	$[Cd]_{worms}$	$[Cd]_{worms}$	$[Cd]_{expected}$	% difference	
			µg/L	%RSD	µg/L	µM	µg/L	Mean
	digestion blank1	Blank	0					
	digestion blank2	Blank	0					
	SRM	SRM	3.6	2.7	10.7		10	7.3
control	500 worms		3.3	3.0	69.6	0.62	0.139	0.142
control	500 worms		3.3	3.2	69.0	0.61	0.138	
control	1000 worms		3.7	1.6	73.7	0.66	0.147	
50µM $CdCl_2$	500 worms		13.8	1.6	299.6	2.67	0.599	0.541
50µM $CdCl_2$	500 worms		14.0	1.1	296.8	2.64	0.594	
50µM $CdCl_2$	1000 worms		21.4	1.5	430.9	3.83	0.431	
50µM $CdCl_2$	OP50 - spiked		154.1	0.6	3148.9	28.0		
